# Personalized Medication for Chronic Diseases Using Multimodal Data‐Driven Chain‐of‐Decisions

**DOI:** 10.1002/advs.202504079

**Published:** 2025-08-11

**Authors:** Xiaoli Chu, Yiheng Ye, Siqiao Tang, Miaoru Han, Guowei Wang, Shuai Lin, Bingzhen Sun, Qingchun Huang, Yan Zhang, Xiaodong Chu, Kun Bao

**Affiliations:** ^1^ State Key Laboratory of Traditional Chinese Medicine Syndrome/Big Data Research Center of Chinese Medicine The 2nd Affiliated Hospital of Guangzhou University of Chinese Medicine Guangdong 510120 China; ^2^ School of Information Science Guangdong University of Finance &Economics Guangdong 510145 China; ^3^ State Key Laboratory of Dampness Syndrome of Chinese Medicine The 2nd Affiliated Hospital of Guangzhou University of Chinese Medicine Guangdong 510120 China; ^4^ School of Electronic and Information Engineering South China University of Technology Guangdong 510000 China; ^5^ Department of Nephrology Zhongshan Hospital of Traditional Chinese Medicine Affiliated to Guangzhou University of Traditional Chinese Medicine Guangdong 528400 China; ^6^ School of Economics and Management Xidian University Xi'an 710071 China; ^7^ Department of Rheumatology The 2nd Affiliated Hospital of Guangzhou University of Chinese Medicine Guangdong 510120 China; ^8^ Research Center of Intelligent Computing and Big Data Technology/ School of Digital Economics Guangdong University of Finance &Economics Guangdong 510145 China; ^9^ Cancer Research Institute/College of Pharmacy/The 1st Affiliated Hospital Jinan University Guangdong 510632 China; ^10^ State Key Laboratory of Dampness Syndrome of Chinese Medicine/Department of Nephrology The 2nd Affiliated Hospital of Guangzhou University of Chinese Medicine Guangdong 510120 China

**Keywords:** chain‐of‐decisions, chronic diseases, multimodal data, personalized medications

## Abstract

The precise matching of medication regimens to individual patients, known as personalized medication, is critical for the effective management of chronic diseases. Traditional machine learning‐based models for personalized medication regimens typically rely solely on either clinical macro‐phenotypes or molecular‐level drug characteristics. It remains challenging to capture the patient‐medication relationship from a comprehensive perspective that integrates individual patient characteristics with macro‐ and micro‐level properties of the medication. Determining patient‐medication relationships constitutes a three‐stage sequential decision process from a clinical decision‐making perspective. Therefore, inspired by Chain‐of‐Thought prompting, which simulates the decision‐making process of human experts, a Multimodal Data‐Driven Chain‐of‐Decisions (MDD‐CoD) framework is proposed, where three‐stage deep learning tasks are sequentially organized to reflect upstream–downstream logical dependencies, thereby forming a coherent clinical decision‐making process. The model incorporates multimodal clinical phenotype data, multi‐attribute medication data, and insights from clinical experts. Performance evaluation of the model involved comprehensive experiments utilizing five datasets covering four chronic diseases sourced from three hospitals. The dataset comprises information from chronic kidney disease (CKD), membranous nephropathy (MN), rheumatoid arthritis (RA), colorectal cancer (CRC), and knee osteoarthritis (KOA), totaling 3173 unimodal, 502 multimodal, and 2187 medication records from 3675 patients. Experimental results demonstrate that the framework achieves enhanced predictive performance in personalized medication decision‐making based on individual patient disease characteristics, surpassing the strongest baseline across all tasks. This framework serves as a foundational model for clinical mixed data, with improved generalization and interpretability in cross‐disease personalized decision‐making tasks. It offers a scalable solution for the implementation of personalized medication regimens for chronic diseases.

## Graphical abstract

A MDD‐CoD is proposed for personalized chronic disease medication. The framework simulates clinical chain‐of‐decisions through three linked deep learning tasks, validated on multi‐center datasets (3 hospitals, 4 diseases), demonstrating enhanced generalization and interpretability in cross‐disease applications.

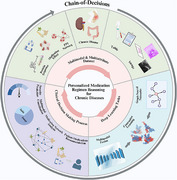

## 1 Introduction

The increasing prevalence of chronic diseases (CDs) presents a significant global health challenge, necessitating effective management as a primary focus for health systems worldwide. Common CDs encompass cardiovascular diseases, respiratory diseases, cancer, diabetes, chronic renal diseases, and arthritis.^[^ ^1^ ^]^ The World Health Organization (WHO) reports that over 70% of global mortality is attributable to CDs.^[^ ^2^ ^]^ In China, over 400 million individuals, representing 28.6% of the total population, have encountered CDs at least once. Furthermore, health‐care costs associated with CDs management constitute over 70% of total expenditures.^[^ ^3^ ^]^ Personalized medication^[^ ^4^, ^5^ ^]^ is the optimal strategy to manage CDs. Currently, one aspect of personalized medications involves designing molecules based on pharmacogenomics and targeting specific patient subgroups for treatment. Another aspect involves adjusting medication dosage, formulation, and medication release methods according to patient needs, disease severity, and stage.^[^ ^6^ ^]^ For example, the development of medication delivery systems^[^ ^7^ ^]^ and the integration of digital health with personalized medicine.^[^ ^8^ ^]^

The clinical decision‐making process for implementing a CDs personalized medication regimen comprises three sequential steps: assessment of the extent of disease considering individual patient differences, combination of therapeutic regimens considering medication properties, and matching of medication regimens considering individual patient characteristics. What constitutes an effective personalized medication regimen is essentially a step‐by‐step process of answering the above three questions. However, due to factors such as the lack of specific indicators in many CDs patients, individual differences, multifactorial pathogenic mechanisms, and the dynamic evolution of diseases, implementing personalized medications for CDs presents a significant challenge.^[^ ^9^ ^]^ The following three areas reflect the difficulties faced by many CDs in establishing personalized medication regimens model. The first area is that the relative weight or ranking of different assessment criteria in clinical conclusions has not been determined, such as laboratory, imaging, and pathologyin clinical conclusions.^[^ ^10^, ^11^, ^12^, ^13^, ^14^ ^]^ This uncertainty has led to significant variability in assessing disease severity of CDs, despite the medical community's efforts to find a uniform definition. The second aspect is the difficulty of identifying thresholds for measuring change to assess the effectiveness of therapeutic measures with traditional assessment methods and baseline model‐based approaches.^[^ ^15^, ^16^, ^17^ ^]^ The third factor is that the most desirable way to use comprehensive diagnostic and treatment data, including pathology, radiology, clinical records, and medication attributes, is to find effective medication regimens that fit the unique characteristics of each patient.^[^ ^18^ ^]^ However, methods to deal with this comprehensive data have not yet been reported.

Methodologies utilizing Chain‐of‐Thought (CoT) and multimodal medical data represent significant advancements in the intersection of intelligent computing and medical research. CoT is a systematic way of thinking that simulates the reasoning and thinking of human experts. CoT directs the model to deconstruct a difficult problem into several sub‐problems through a sequence of coherent phases, illustrating the concepts and logical connections of reasoning, progressively resolving the issue, and culminating in the final solution.^[^ ^19^ ^]^ CoT‐based models enhance interpretability by generating a step‐by‐step reasoning sequence, providing clear insight into the model's cognitive process.​ It has garnered considerable attention in disease diagnosis, including early detection of sepsis,^[^ ^20^ ^]^ assessment of stroke severity,^[^ ^21^ ^]^ identification of infectious disorders,^[^ ^22^ ^]^ pathological categorization,^[^ ^23^, ^24^ ^]^ echocardiographic classification,^[^ ^25^ ^]^ and molecular mechanisms of COVID‐19.^[^ ^26^ ^]^ Data‐driven modeling research in medicine^[^ ^27^, ^28^ ^]^ increasingly tends to integrate different modalities, such as imaging,^[^ ^29^ ^]^ electronic health records,^[^ ^30^ ^]^ laboratory test metrics,^[^ ^31^, ^32^ ^]^ and bioinformatics data.^[^ ^31^, ^32^ ^]^ Integration of models from different modalities can reveal heterogeneity of complex diseases and further combine medication micro‐molecular data^[^ ^33^, ^34^ ^]^ to tailor healthcare and improve personalized medicine. Innovations in research methods include multimodal information learning classification methods,^[^ ^35^ ^]^ fusion strategies,^[^ ^36^ ^]^ clinical validation and interpretable methods.^[^ ^29^ ^]^ These methods are used to address the heterogeneity, integration complexity, and interpretability of multi‐source healthcare data.^[^ ^37^ ^]^ Data‐driven models under CoT have demonstrated significant advantages from different perspectives, such as predicting disease progression and optimizing medication regimens, provding a critical foundation for quantifying personalized medication regimens for CDs. However, a common limitation of existing approaches is that these methods have fail to establish a chain relationship from disease macro‐phenotypes to micro‐medication properties, which does not allow for accurate personalized medication for CDs. To overcome this challenge, new, more comprehensive clinical phenotype and medication micro‐property datasets and methods are needed to improve the accuracy of clinical decision‐making. At the same time, it allows for reasoning and recommendation of medication regimens that are tailored to individual patient characteristics.

In this study, inspired by the progressive decision‐making advantages of CoT and multimodal deep learning, a Multimodal Data‐Driven Chain‐of‐Decisions (MDD‐CoD) framework is designed to address personalized medication for CDs. Specifically, we decompose the complex task of personalized medication regimens into a stepwise decision process from a holistic full‐cycle perspective of disease management. Accordingly, we propose a Chain‐of‐Decisions (CoD) that sequentially integrates three key components: modeling individual patient characteristics, identifying effective drug combinations, and matching patient profiles to personalized medication regimens. CoD encompasses three core decision‐making stages in personalized medication planning: disease severity assessment, therapeutic regimen selection, and personalized medication decision‐making. Furthermore, we introduce a multimodal data‐driven deep learning approach that integrates two critical dimensions of personalized medication research: patient‐specific disease characteristics and the pharmacological properties of both ​herbal medicines​​ and conventional drugs. In this design, patient medication data serve as the bridging medium, enabling the construction of three sequentially linked deep learning tasks that mirror the logic of clinical decision‐making: first, multimodal diagnostic data are fused to assess disease severity; next, multi‐attribute drug combinations are evaluated for therapeutic efficacy; and finally, the optimal regimen is inferred by matching patient characteristics with candidate medications. These tasks are organized in an upstream–downstream dependency hierarchy​​, forming a coherent decision chain that explicitly simulates the real‐world clinical reasoning process. Through this framework, personalized treatment regimens can be generated based on disease severity and the patient's ​​integrated​​ clinical profile, providing more robust support for clinical decision‐making in the management of CDs.

A comparative analysis with the strongest baseline of deep learning algorithms demonstrates the superior performance of our approach in the prediction of patient‐medication matching. Furthermore, in the multimodality of imaging, pathology, and laboratory test indicators, the experimental results highlight the key role of histopathology in the diagnosis of colorectalcancer (CRC) and chronic kidney disease (CKD), while laboratory indicators play a key role in the diagnosis of rheumatoid arthritis (RA), relative to other modalities. Meanwhile, for CDs, experiments have shown that multiple attributes of medications can better predict the efficacy of medication combinations relative to individual attributes. Our framework is a foundational model that explicitly incorporates the clinical decision‐making process for ​​CDs​​, integrating heterogeneous real‐world data. It demonstrates strong generalization in ​​cross‐disease personalized diagnostic and therapeutic decision‐making​​, providing ​​healthcare professionals​​ with accurate clinical decision support. Specifically, the main contributions of our work are as follows:

(1) We leveraged the progressive decision‐making paradigm of CoT to personalize clinical decisions for CDs, by integrating patient needs, disease severity, and pharmacogenomic bioinformatics. To achieve this, we developed a CoD framework, which systematically divides the clinical decision‐making process into three stages with clear logical relationships. Furthermore, we introduced CoD to enhance the interpretability of deep learning models for personalized medication decision‐making.
(2) We constructed three deep learning tasks with chain‐like relationships for the three stages of personalized medication for CDs. These tasks are integrated within a ​​MDD‐CoD model, bridging clinical diagnosis and treatment. This model integrates patient disease characteristics, medication characteristics, pharmacological information characteristics, and patient medication regimens to improve the accuracy of personalized medication predictions for CDs.
(3) We tested MDD‐CoD on our proprietary multimodal comprehensive dataset. We utilized five datasets covering four CDs from various hospitals, which are CKD, membranous nephropathy (MN), RA, CRC, and knee osteoarthritis (KOA), totaling 3173 unimodal, 502 multimodal, and 2187 medication records from 3675 patients. The experimental results demonstrate that MDD‐CoD possesses superior capabilities in clinical incremental decision‐making for personalized medication regimens. It also highlights the generalizability of the model in personalized medicine for CDs.

## 2 Result

### 2.1 Overview of the MDD‐CoD Model

**Figure 1:**
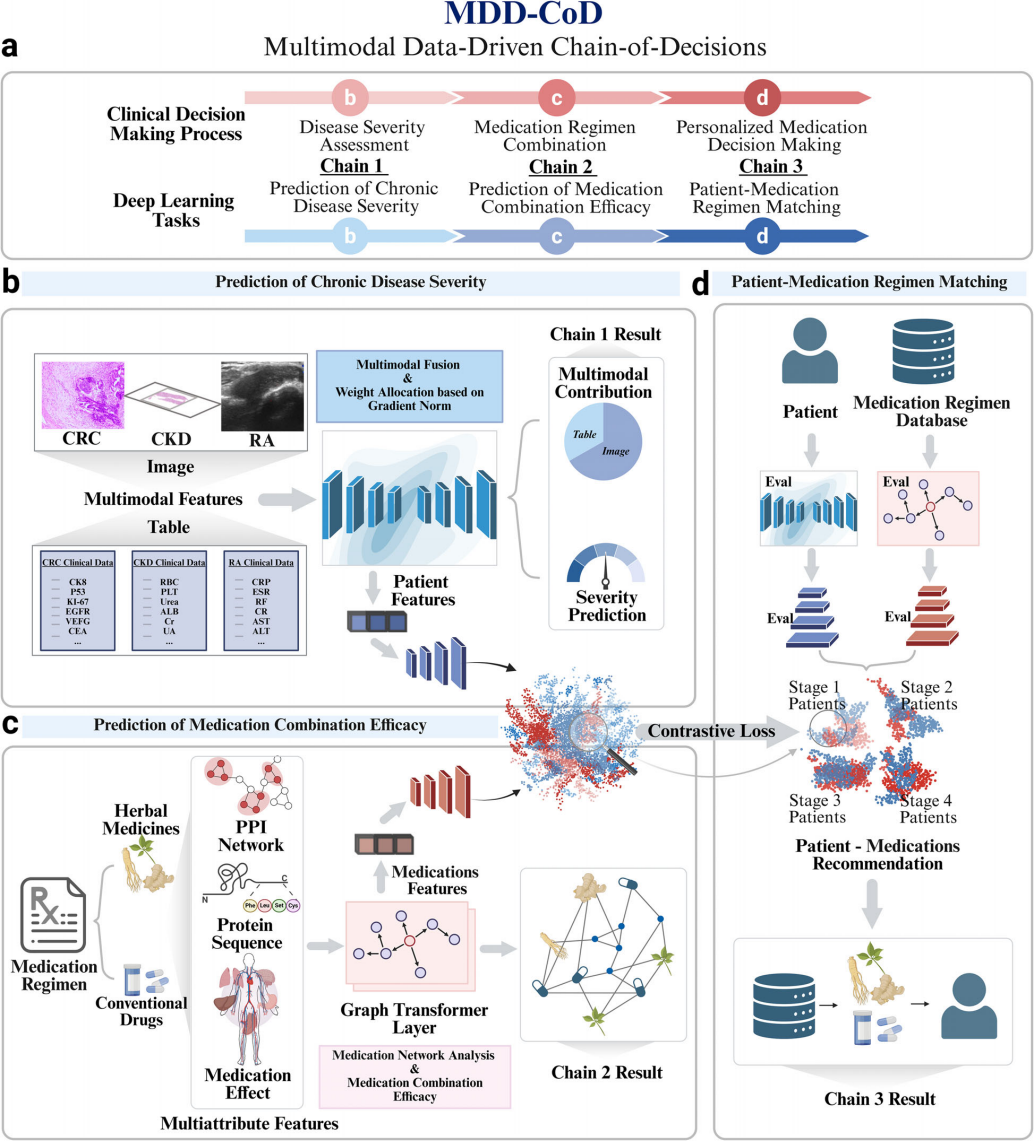
Overview of the MDD‐CoD model. MDD‐CoD integrates patient multimodal diagnostic data with medication multi‐attribute data by constructing chained relationships between individual patient characteristics and medication regimens. MDD‐CoD performs well in all baseline models. MDD‐CoD was divided into three tasks. a) Prediction of CDs severity. Aiming at the multimodal clinical phenotype information of patient's imaging, histopathological, and laboratory indicators, the CDs severity prediction model was constructed to solve the problem of the relative weights of different modalities in the disease assessment decision. b) Prediction of medication combination efficacy. Considering the complex relationship when multiple medications, such as herbal medicines and conventional drugs are used in combination, a graph neural network model is constructed. Where the graph nodes are medications and the edges are medication combinations, a priori knowledge graphs. The node features include multi‐attribute information consisting of protein networks, protein sequences, indications, and side effects. MDD‐CoD will construct medication features from the graph to predict the severity labels of the corresponding patients, which will be used to accurately learn the mechanism of action when medications are used in combination. c) Patient‐medication regimens. d) MDD‐CoD pairs the patient's clinical characteristics and medication characteristics through comparative learning to give a ranking of effective medication regimens for the patient's individual characteristics. When predicting medication use for new patients, the patient's individual characteristics will be matched with the existing medication characteristics to achieve individualized medication‐assisted decision‐making.

**Figure** **1** outlines the MDD‐CoD model, which represents the underlying framework for personalized medication regimen decision‐making for CDs. We introduced the CoD, chaining two intermediate decision processes that have a logical relationship with the personalized medication regimen. For these three processes, disease severity assessment, medication regimen selection, and personalized medication regimen decision‐making, three corresponding deep learning tasks are constructed, as shown in Figure 1a.

First, to accurately assess the severity of CDs, the primary issue is to determine the decision weights of different classification criteria in clinical diagnostic conclusions, such as laboratory criteria, imaging criteria, and pathology criteria. Therefore, a multimodal assessment model for diagnostic data fusion is constructed oriented to the multimodal clinical phenotypic information of the patient's imaging, pathology, and laboratory indicators. The weight of each modality in the diagnostic decision is derived by dynamically adjusting the contribution of the classification loss function of a single modality to the total loss function. This model is shown in Figure 1b. Specifically, a gradient‐based dynamic adjustment strategy is introduced as a means of obtaining the contribution of different modalities to the final prediction result. This strategy is a training process that generates modal weights for which multiple iterations of the multimodal model are required to determine the optimal performance. This process simulates the decision‐making process of a physician dynamically adjusting the weights of multiple sources of diagnostic information to predict or optimize assessment conclusions.

Second, to more comprehensively predict the efficacy of medications in combination, a heterogeneous graph‐based model was constructed to predict the efficacy of multi‐attribute medication combinations, from the macroscopic attributes of the medications to the microscopic molecular level, as shown in Figure 1c. Specifically, the frequency of co‐occurrence of different medication pairs served as a priori knowledge to construct the initial medication graph structure for a single patient. Within the graph, medications were the nodes of the graph, and medication features such as the four natures, five flavors, and meridian tropism of herbal medicines, protein sequences, side effects of conventional drugs, and medication targets were the features of the nodes. The node features of the initial graph structure were reconstructed using a graph neural network based on the attention mechanism. The reconstructed nodes could better characterize the relationships between medications, such as complementarity and exclusivity, and thus help to match individual patient characteristics with medication characteristics.

Finally, MDD‐CoD reasons about the matching relationship between patient and medication regimen by learning the patient's clinical features in comparison with the medication features, as shown in Figure 1d. It projects the two sets of features into a shared embedding space and characterizes the degree of matching degree clinical and medication features in a unified semantic space. When a medication recommendation is made for a new patient, the patient's characteristics will be matched with the existing medication features for patient–medication matching, thus realizing personalized medication‐assisted decision‐making.

### 2.2 Matching on Patient‐Medication Regimens for Chronic Diseases

**Figure 2:**
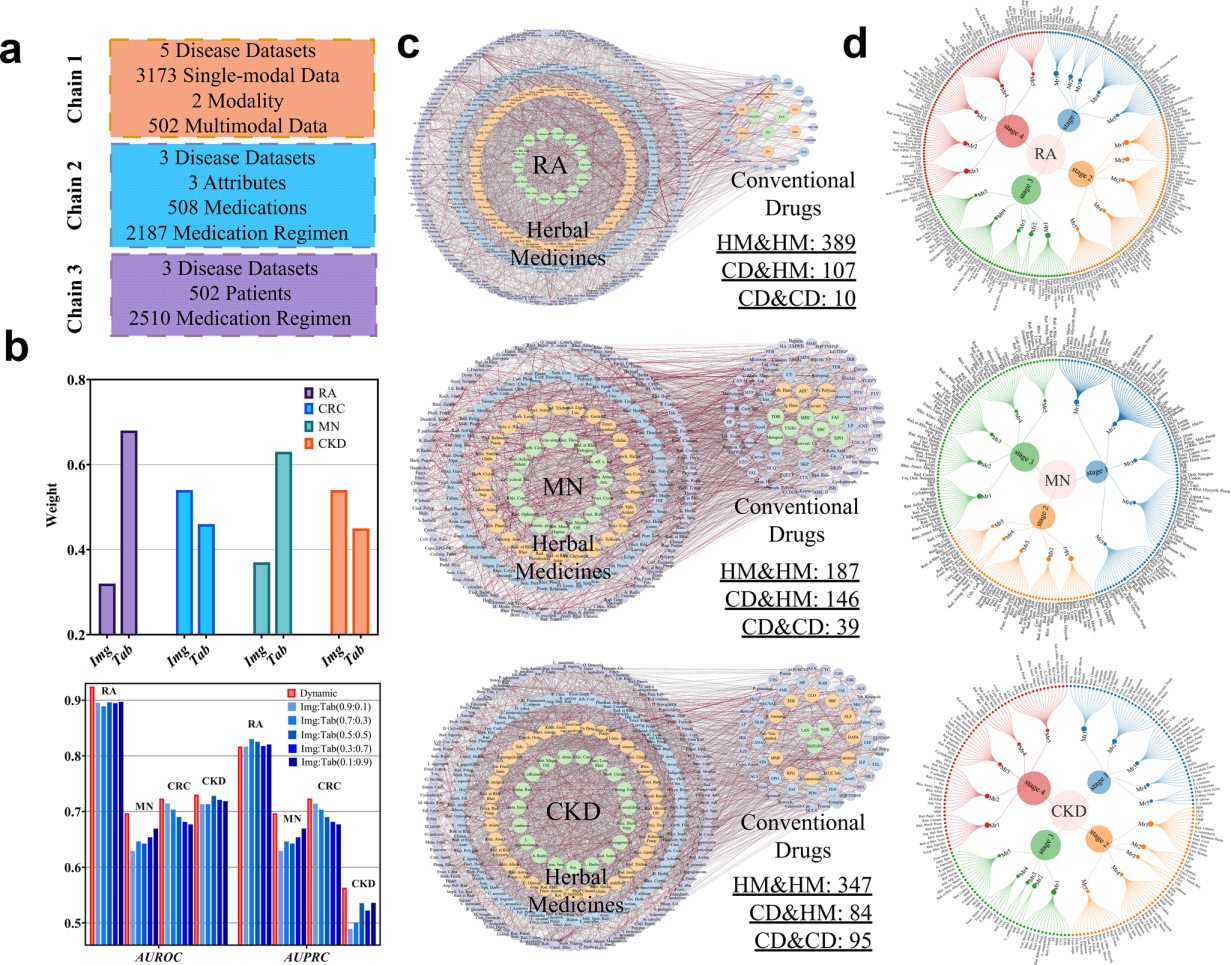
Clinical outcomes of personalized medication strategies for CDs. a) Our methodology utilizes diagnostic and medication data to make sequential clinical decisions, ultimately deriving personalized medication regimens that are precisely matched to each patient's disease characteristics. b) A multimodal diagnostic data fusion assessment model is utilized to determine the weights of multiple examination modalities in disease assessment decisions. The results demonstrate that the decision‐making strategy of dynamic weighting is effective in CRC, MN, RA, and CKD disease assessment decisions. c) The combination of herbal and conventional drugs is predicted to be more common. Nodes indicate individual medication and edges indicate combinations of medications. Larger circles indicate herbal medications, and smaller circles indicate conventional drugs. Larger nodes indicate better efficacy of individual medications, and darker edges indicate better efficacy of medication combinations. The results indicate that the efficacy of an individual medication is not representative of its efficacy when combined with other medications. d) These three figures represent the patient‐medication matching results for three CDs. Under the same classification of disease severity, multiple medication regimens are recommended for each patient's individual characteristics. In an assisted‐support clinical decision‐making application, physicians can choose again among the recommended regimens based on their experience or preference. Details of medication abbreviations are provided in Appendix 6.

As shown in **Figure** **2**a, we use five CDs datasets(RA, CRC, CKD, MN and KOA) to chain together decision‐making processes, disease severity assessment and medication combinations, and ultimately reason to draw conclusions on patient‐medication regimen matching for CDs. In the diagnostic and therapeutic decision‐making of CDs, such as CRC, pathological assessments and laboratory indicators serve as key foundations for clinical staging and prognosis prediction. Specifically for CRC, pathological staging directly informs treatment planning, while laboratory indicators—such as carcinoembryonic antigen and inflammatory markers—dynamically reflect disease progression and the patient's systemic condition.^[^ ^38^ ^]^ Therefore, we integrated the bimodal information from images and tablesto simulate the comprehensive decision‐making logic of clinicians, which is qualitative pathology combined with quantitative indicators. The four diagnostic datasets(RA, CRC, CKD, and MN), covering both image and tabular modalities, are used to train and validate a model for multimodal diagnostic data fusion to solve the first sub‐problem in MDD‐CoD, the assessment of disease severity. Furthermore, the relative weighting of the information gained from the various testing techniques in clinical diagnosis. In addition, KOA is a unimodal tabular dataset that is used to train and validate model performance assessment under unimodal data conditions and to compare it with multimodal performance. As MN is the most common cause of nephrotic syndrome in adults and has a large sample size (72 cases) in the CKD dataset, it is independently assessed for disease labeling and was subjected to separate experiments and analyses. As shown in Figure 2b, our study reveals the modality‐assigned weights for the four categories of RA, CRC, CKD, and MN. In the multimodality of image data (imaging, pathology) and tabular data (laboratory indicators), the experimental results emphasize the key role of pathology in the diagnosis of CRC, CKD, and MN, whereas the laboratory indicators play a key role in the diagnosis of RA, relative to the other modalities. Among them, the clinical guidelines for CRC clarify the importance of pathological investigations for diagnosis and treatment,^[^ ^39^ ^]^ and its pathological modalities are representative of the important decision‐making basis in CDs.

In addition, we used the fixed weights fusion method and indicate that fixed weight assignment is difficult to accommodate differences in modal importance under different diseases, and unimodal weighting errors are transmitted to the final prediction through fixed weights. In contrast, the method using dynamic weighting with multimodal contributions can automatically adjust the weights according to the contribution of each modality to the prediction of disease severity, thus better integrating the information of different modalities. Physicians who are able to dynamically adjust the weights of the examination modalities have higher accuracy in their assessment results, relative to the situation where the weights of the examination modalities are fixed throughout the disease assessment decision‐making process. When new patients are added to the dataset, the dynamic adjustment strategy of the model is able to update the weights of the criteria for assessing disease severity in a timely manner, providing great potential for personalized patient diagnosis and treatment, as shown in Figure 2b. This seminal finding clarifies the contribution of multi‐source diagnostic tools in the final clinical conclusion and gives examples for the allocation of weights to classification criteria for other CDs in clinical decision‐making.

To analyze the principle of prescription by clinical experts, we established a dataset of medication regimens for three CDs(RA, CKD, and MN), based on the attributes of the macro‐level pharmacology of the medications, the micro‐level protein interaction relationships of the medications, and the protein sequences, followed by the construction of a prediction model for the efficacy of the medication combinations, as shown in Figure 2c. The macro‐pharmacology of medications includes the natures, flavors, meridian tropism, and efficacy of herbal medicines, as well as the side effects of conventional drugs. We have ranked the efficacy of the two‐medication combinations by using the medication combination efficacy prediction model, and the experimental results show that the pattern of medication varies from disease to disease in the treatment of CDs. We selected the combination of medications that ranked in the top 15% of the efficacy outcome values for analysis. We found that herbal combinations dominated in RA treatment. In MN treatment, traditional herbal prescriptions, alone or combined with conventional drugs, are commonly used, highlighting the need for integrative medicine. In CKD, the regimen was dominated by conventional medications, which is more frequent than that in RA and MN.

Additionally, the combination of herbal and conventional medications has demonstrated superior therapeutic efficacy. Specifically, the two–two combinations of RA medications, *Hedyotis diffusa* and Zhengqing Fengtongning extended‐release tablet, receive the highest rankings. In MN, Panax notoginseng and Rabeprazole rank the highest in the two‐two combination of medications. Methylprednisolone and Pinellia ternata rank the highest among the two combinations of CKD medications. This may imply that herbal medicines could relieve the side effects of conventional drugs. For example, the modulating effect of Panax notoginseng on the level of intestinal microorganisms and the gastroprotective effect of rabeprazole may be clinically suggestive of reducing the gastrointestinal adverse events of methylprednisolone.^[^ ^40^ ^]^

The research on the previous two sub‐problems successfully completed the extraction of multimodal clinical phenotype and multi‐attribute medication regimen features. Building on these foundations, to further derive medication regimens corresponding to the differential features of individual patients, we match patient characteristics with medication features and generated a ranked list of recommended regimens based on individual characteristics. We collect three datasets of CDs (RA, MN, and CKD), easch containing complete patient features and corresponding medication regimens. Under the same disease severity classification criteria, 5 medication combinations are recommended for each patient, as shown in Figure 2d. As an example, the patient with MN, hypertension and anemia in Figure 2d, referred to as the reference individual, is taking medication for MN along with Irbesartan and a polysaccharide–iron complex to treat hypertension and improve anemia. Figure 2d presents the 5 medication regimens under different severity levels in the reference individual. When predicting herbal medication regimens in combination with conventional drugs for a new patient with MN with hypertension and anemia, multiple medication combination regimens are given by calculating the similarity of diagnostic information between the new patient and the reference individual. Then, based on their experience or preference, physicians make a final selection among the recommended regimens according to patient‐specific characteristics, such as medication resistance, adverse reactions, and other clinically relevant factors.

### 2.3 Predictive Performance of MDD‐CoD

Compared with the baseline model, MDD‐CoD shows excellent performance. The innovative approach of the model introduces the integration of CoD with the multimodal deep learning model of clinical diagnosis and treatment, which utilizes the multimodal features of diagnostic information and the multi‐attribute information of medication. The multimodal features of diagnostic information include pathological images and laboratory data. The multi‐attribute information of medication includes natures, flavors, meridian tropism, efficacy, side effects, protein sequence, protein–protein interaction (PPI) networks, and priori knowledge graphs. It simulates the decision‐making process of clinical experts in reasoning the medication regimen based on the condition by chaining the decision‐making process related to personalized medication regimens for CDs and deeply investigates the principle of matching individual patient characteristics with medication characteristics. This comprehensive approach ensures high accuracy across various CDs prediction tasks, highlights a full‐chain information integration model for CDs diagnosis and treatment, and demonstrates a holistic understanding of personalized medicine in CDs management.

**Figure 3:**
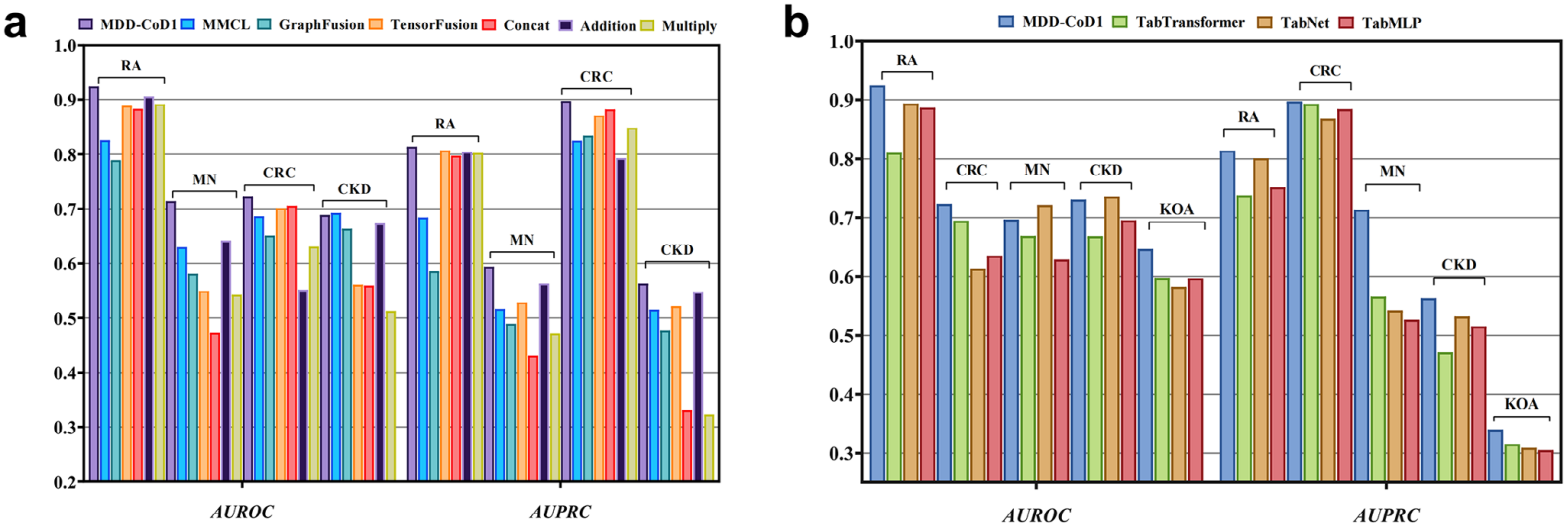
Performance evaluation of MDD‐CoD1 on the test set. a) MDD‐CoD1 effectively integrates multimodal disease characteristics of patients and outperforms all baseline models in predicting disease severity. b) The FT‐Transformer significantly outperformed baseline models in predicting CDs severity across both unimodal (KOA) and multimodal (RA, CRC, MN, CKD) datasets, with multimodal inputs showing superior diagnostic performance.

**Figure 4:**
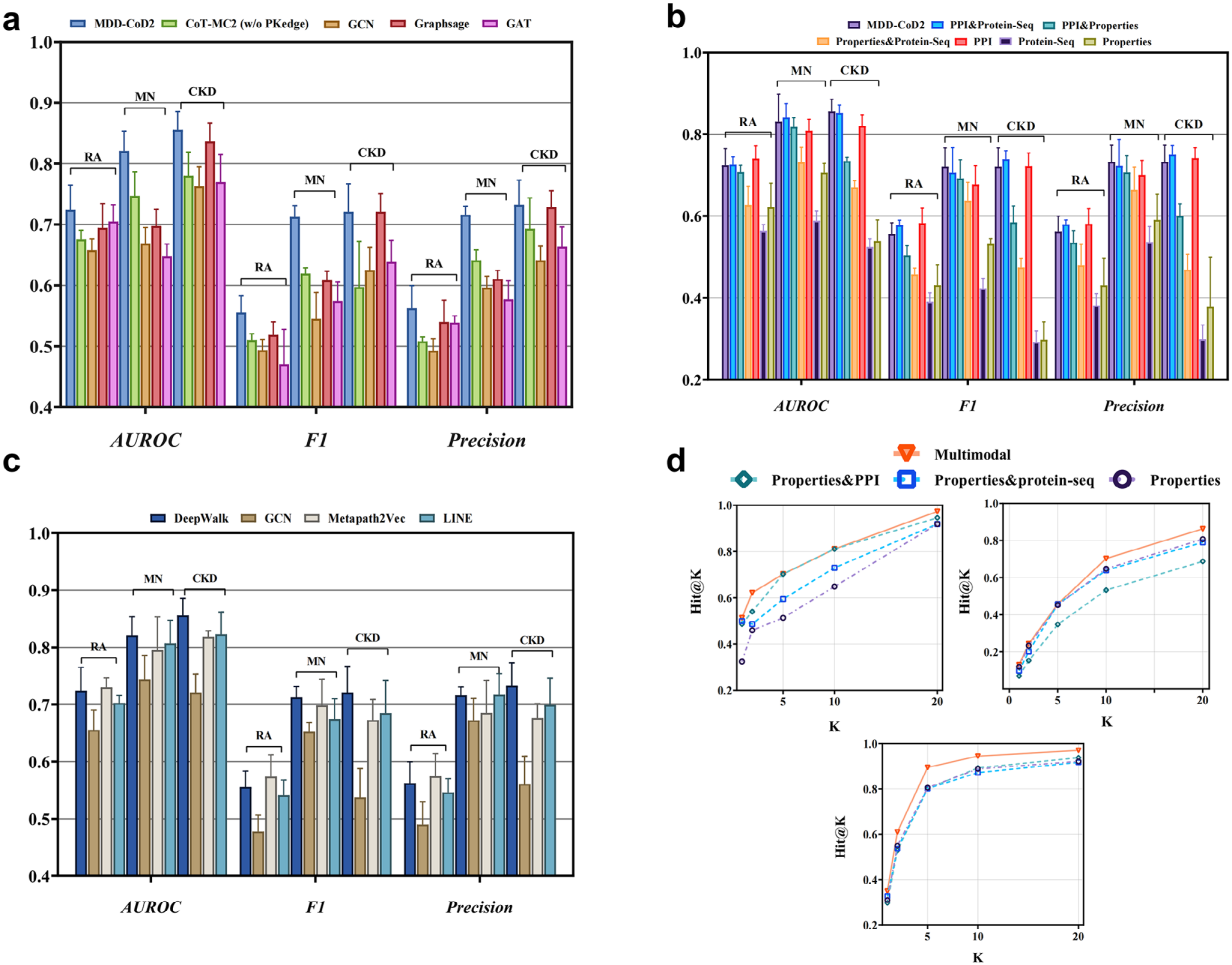
Performance evaluation of MDD‐CoD2 and MDD‐CoD3 on the test set. a) MDD‐CoD2 provides an in‐depth characterization of how macro‐ and micro‐level medication attributes influence medication–medication interactions, with its knowledge‐enhanced graph transformer architecture outperforming all baseline models. b) Multiattribute feature fusion effectively addressed the inherent limitations of unimodal analysis through complementary integration, with performance progressively improving as more comprehensive medication attributes were incorporated. c) Deepwalk outperformed other commonly used graph embedding methods on biological data across all evaluation metrics by employing short random walks that covered the majority of protein nodes. d) In MDD‐CoD3, multiattribute medication features more effectively supported optimal regimen selection, highlighting the model's strong capability in personalized therapeutic decision‐making for CDs.

MDD‐CoD connects three deep learning tasks (MDD‐CoD1, MDD‐CoD2, and MDD‐CoD3) together, as shown in **Figures** **3** and **4**. The MDD‐CoD model demonstrates superiority in comparison with various baseline methods on all three tasks, as shown in Figures 3a and 4a,d. The deep learning approach based on the fusion of multimodal diagnostic information, including the first task MDD‐CoD1, specifically emphasizes a deep learning approach with multimodal information such as images and laboratory indicators. The method for disease severity assessment, with comprehensive consideration of features related to images and laboratory indicators, provides a broader understanding of CDs state assessment. The baseline model related to multimodal data includes the SOTA multimodal fusion framework,^[^ ^41^ ^]^ the classical multimodal fusion module(Graph Fusion,^[^ ^42^ ^]^ Tensor Fusion^[^ ^43^ ^]^), and the underlying feature fusion operations (concat, addition, multiply). The experimental results in Figure 3a indicate that MDD‐CoD1 effectively integrates patient's multimodal features and outperforms the baseline models in predicting disease severity with satisfactory results. The four datasets outperform the optimal baseline by an average of 2.6% on the AUROC metrics and by an average of 1.7% on the AUPRC metrics.

The MDD‐CoD1 experiment is shown in Figure 3b. We find that FT‐Transformer can demonstrate superiority when predicting single‐modal data and being integrated into the multimodal fusion model as a multimodal feature encoder. In the single‐modal KOA dataset, the multimodal RA, CRC, and MN datasets, all evaluation indicators exceeded the baseline models. Furthermore, the predictive indicators on the multimodal dataset are significantly better than those on the single‐modal dataset, which also indicates that multimodal medical data can assist in diagnosis more accurately.

The deep learning model for predicting the efficacy of medication combinations is a knowledge augmentation‐based graph neural network MDD‐CoD2. We introduced a priori knowledge mapping of medication combinations for the medication dataset to construct a multiattribute graph structure. This approach offers a clear characterization of the effects of macro‐ and micro‐medication attributes on medication combination patterns. MDD‐CoD2 is compared with relevant baseline models, including the classical architecture GCN,^[^ ^44^ ^]^ GraphSAGE,^[^ ^45^ ^]^ the attention‐driven model GAT,^[^ ^46^ ^]^ and a fixed‐weight control model (w/o PKedge). Experiments in Figure 4a have shown that MDD‐CoD2, focusing on the prediction of medication combination efficacy through a priori knowledge‐driven Graph Transformer architecture, has achieved satisfactory results. This is because MDD‐CoD2 incorporates more comprehensive medication macro‐pharmacology (natures, flavors, meridian tropism, efficacy, and side effects of conventional drugs), medication micro‐protein interaction relationships, protein sequences, and other multiattributes of medications. These multiattributes are integrated into the medication features of the nodes, allowing Transformer to more accurately aggregate and reconstruct the multiattribute features of medications when performing attentional computations. The richer characterization information given to each medication node can improve the efficacy prediction results for the medication combinations. In three datasets, AUROC, F1, and Precision improved by 3.1%, 4.3%, and 3.2% on average.

Another noteworthy observation in the experiments on predicting medication combination efficacy is that unimodal analysis based on PPI exhibited significant advantages, whereas single protein sequence features and pharmacological features showed poor predictive performance, as shown in Figure 4b. The average prediction performance of the unimodal fusion of PPI in AUROC and the F1 metrics of RA was optimal. Multiattribute fusion effectively overcomes the inherent limitations of unimodal analysis through feature complementarity, and the predictive performance of the model is further improved in some datasets after fusing single Protein‐Seq features and Properties features with PPI features. Among them, in the dual‐property fusion task of PPI combined with Protein‐Seq, the average prediction performance is optimal in the AUROC metrics of MN, F1, and Precision metrics of RA and CKD. In the three‐attribute fusion task of PPI combined with Protein‐Seq and Properties, the average prediction performance is optimal in the AUROC index of CKD and the F1 and Precision indices of MN. It is worth noting that most of the three assessment metrics (AUROC, F1, and Precision) of the three‐modal model stably exceeded the unimodal optimal values for all datasets in the four‐fold cross‐validation, and the different medication information had a significant impact on the prediction results of patient‐medication matching. The gradual improvement of performance after considering the multiattribute features of medications in a comprehensive manner also suggests that the multiattribute features of medications can more effectively help patients select effective medication regimens. Notably, in the feature extraction of PPI attributes, Deepwalk covers most of the protein nodes by short random wandering, which outperforms other graph embedding methods (GCN, Metapath2Vec,^[^ ^47^ ^]^ LINE^[^ ^48^ ^]^) commonly used for biological data in all evaluation metrics, as shown in Figure 4c.

Finally, we perform a comprehensive validation of the MDD‐CoD model on three disease datasets (RA, MN, and CKD) with complete modalities by performing a CLIP‐based cross‐modal alignment computation of individual patient and medication features. The experiments in Figure 4d indicate that the introduction of multimodal features of diagnostic information and multiattribute features of medication enables the model to achieve superior prediction performance in the patient‐medication matching task, reaching 94%, 81%, and 70%, respectively, in HIT@10, which confirms the validity of matching medication profiles with patient features.

### 2.4 Ablation Experiment

To further validate the effectiveness of MDD‐CoD, we conducted additional ablation experiments on four disease datasets, and the results are shown in **Table** **1**. The ablation experiments were conducted on various modules in the MDD‐CoD task, including the effects of modal features, weight dynamic adjustment strategies, modal fusion, and multiple attributes of medications on the predictive performance of the model. The results in Table 1 clearly show that considering only unimodal features leads to a degradation in the predictive performance of the disease severity model, further emphasizing the need to incorporate multiple examination modalities and information when assessing CDs severity. However, it is worth noting that in the CRC dataset, a single image modality did not pull away from the prediction results of multiple fusions, and thus a single pathology image is also of great reference value in the subsequent actual clinical diagnosis of CRC.

**Table 1:** Ablation experiment on the four datasets of the multimodal dynamic weighting model (MDD‐CoD1). MI (Medical Image), TD (Tabular Data), GNW (Grad‐Norm Weight), CA (Crocs Attention).

MI	TD	GNW	CA	CRC		MN		RA		CKD	
				AUROC	AURPC	AUROC	AURPC	AUROC	AURPC	AUROC	AURPC
✓	×	×	×	0.6703	0.8451	0.5041	0.3837	0.5172	0.4466	0.5043	0.4324
×	✓	×	×	0.5407	0.6819	0.5931	0.4157	0.7735	0.4493	0.5724	0.4964
✓	✓	×	×	0.6967	0.8838	0.5874	0.6289	0.9255	0.8255	0.6780	0.5354
✓	✓	✓	×	0.7033	0.8908	0.6654	0.6234	0.9112	0.7662	0.6938	0.5480
✓	✓	✓	✓	0.7231	0.8977	0.6968	0.7135	0.9244	0.8137	0.7304	0.5630

To verify the performance of the cross‐attention mechanism in fusing features from both modalities, the cross‐attention mechanism is replaced with a simple feature fusion method in the ablation experiments. The experimental results show that the cross‐attention mechanism is more generalized and has the best average prediction accuracy across the dataset. The collaboration of the gradient‐paradigm‐based weight adjustment design and the cross‐attention‐based multimodal fusion module can effectively integrate multimodal features and achieve superior prediction performance.

## 3 Discussion

This study introduces CoD concept to develop MDD‐CoD, a multimodal data‐driven deep learning model that emulates the decision‐making process of clinical experts in disease diagnosis and treatment. By sequentially linking two interrelated clinical decision‐making processes, disease severity assessment and medication regimen combination, we establish a reasoning paradigm for personalized medication decision‐making in CDs. We emphasize these dual decision processes to enhance the precision of medication recommendation. MDD‐CoD integrates clinical phenotypic features (including imaging, pathology, and laboratory indicators) with medication characteristics (encompassing priori knowledge graph of medication combinations for both herbal and conventional drugs, protein networks, protein sequences, indications, and side effects). This work pioneers a methodological framework for personalized medication decision‐making in CDs, with experimental results strongly validating its efficacy. Notably, our findings demonstrate that dynamic adjustment of weighting coefficients across diagnostic modalities significantly improves the accuracy of disease assessment compared to fixed‐weight approaches, a breakthrough with transformative implications for personalized diagnosis and evaluation in CDs management. Through systematic integration of chronic clinical diagnostic/treatment workflows and associated datasets, MDD‐CoD enables patient‐specific disease evaluation strategies, optimized medication combination strategies, and ultimately personalized medication recommendations based on individual characteristics of CDs patients.

While our method demonstrates promising performance in personalized treatment recommendation for CDs, several limitations remain. Our model has yet to incorporate more microscopic omics data (e.g., genomics, proteomics, metabolomics data), as comprehensive data‐driven approaches could better identify genotype‐phenotype associations and risk factors within CDs subpopulations, thereby advancing precision medicine in CDs management.^[^ ^28^ ^]^ Another critical consideration lies in the generalizability of our approach across other categories of CDs. Current framework parameters were derived from musculoskeletal disorders (KOA), autoimmune disease(RA), renal disease (CKD), and cancer (CRC), without encompassing all subtypes of CDs. Future efforts will focus on expanding disease coverage (e.g., cardiovascular, metabolic, and respiratory systems), and enhancing dataset diversity by incorporating a broader range of patient characteristics and medication attributes. In addition, implementing transfer learning methodologies could enable parameter adaptation to novel diseases, thereby improving the robustness of personalized therapeutic recommendations and ensuring the scalability and adaptability of the framework across heterogeneous CDs types.

Additionally, our method focuses on personalized medication recommendation under multimodal diagnostic conditions. Notably, achieving accurate medication regimen recommendations using single‐modality data holds significant importance for avoiding excessive medical interventions. For instance, whether a medication regimen can mitigate the deterioration of imaging or histopathological features in CDs patients, specifically, its capacity to halt progressive organ or tissue degeneration, serves as a critical indicator for evaluating therapeutic efficacy. However, our current dataset lacks temporal imaging or histopathological data. To address this limitation, future research will prioritize the integration of temporal multimodal data sources with pre‐trained models. This expansion aims to enhance the alignment between CDs progression and therapeutic strategies, ultimately establishing a more precise framework for personalized medication recommendation in CDs management.

## 4 Related Work

Accurate identification of patient‐specific characteristics and elucidation of medication combination principles constitute two pivotal decision‐making steps in recommending personalized medication regimens for CDs. Various multimodal fusion‐based decision‐making approaches have been developed, including:

***Chain‐of‐Thought reasoning model***. Currently, CoT research in healthcare primarily utilizes structured and unstructured data, employing hierarchical CoT reasoning to derive medical conclusions through multi‐generational integration.^[^ ^21^ ^]^ The underlying principles and intrinsic mechanisms of CoT represent a critical research direction, where the thought chain is conceptualized as a multi‐step composite function.^[^ ^49^ ^]^ While CoT provides intermediate reasoning steps, existing methods heavily depend on instructional prompts, and the generation process of thought chains lacks comprehensive interpretability.^[^ ^50^ ^]^ Furthermore, challenges persist in modeling cross‐modal interactions, making multimodal reasoning chains a significant unsolved problem.

***Multimodal fusion and predictive model***. A multimodal disease recurrence management system was developed by integrating traditional clinical factors with novel molecular biomarkers.^[^ ^51^ ^]^ Additionally, multimodal architectures combining histopathological images and gene expression data for patient survival prediction have advanced deep learning in multimodal healthcare analytics.^[^ ^31^ ^]^ Other studies leverage routine blood tests, clinical data, and multi‐omics data to predict therapeutic benefits^[^ ^52^ ^]^ and risks.^[^ ^53^ ^]^ These multimodal fusion approaches predominantly focus on integrating two modalities (e.g., text, images, numerical data, network graphs) rather than addressing the complexity of three or more modalities.

***Graph neural network based medication mechanism analysis***. Numerous studies employ medication–medication interactions, biomedical knowledge graphs, molecular profiles, and patient characteristics to predict medication efficacy using GNNs and their variants.^[^ ^36^, ^54^, ^55^, ^56^ ^]^ Network medicine frameworks have also been applied to analyze topological relationships between herbal medication targets and disease phenotypes.^[^ ^57^ ^]^ While these methods explore medication–medication and medication–disease interactions, they do not explicitly account for interactions between patient‐specific features and medications.

***Personalized medication regimen decision‐making***. Most studies treat therapeutic outcomes as labels for personalized medication recommendation via patient‐clinical feature matching. For instance, a small‐data clinical decision support system for personalized medication dosing is developed.^[^ ^58^ ^]^ Predicted patient‐specific medication efficacy by aligning clinical data with known therapeutic profiles.^[^ ^59^ ^]^ Recent advancements include using demographic features to forecast adverse medication reactions^[^ ^56^ ^]^ and leveraging disease progression sequences (e.g., disease/surgical histories) with knowledge graphs for medication set recommendation.^[^ ^60^ ^]^ Although these methods enable medication recommendation, they predominantly focus on medication‐medication interactions rather than comprehensively incorporating disease‐specific characteristics.

Existing studies have proposed pathways and methods for personalized therapeutic recommendations in CDs from diverse perspectives, emphasizing the importance of multi‐source data in precision medicine. However, these works predominantly focus on either clinical data or medication information, failing to holistically model the chain‐like relationships spanning from patient‐specific characteristics to pharmacological attributes. Alternatively, some approaches reduce medications to patient labels while neglecting their intrinsic feature‐specific information. Our study addresses this limitation by integrating four modalities, text, numerical data, images, and protein network structures, to construct a framework that systematically links patient‐specific characteristics with medication information, thereby resolving the challenge of personalized medication regimen decision‐making in CDs.

## 5 Conclusion

We have established and validated a robust MDD‐CoD model for CDs management. This model leverages a novel dataset encompassing four diagnostic and therapeutic scenarios, explicitly producing stepwise decision‐making processes that delineate disease–medication matching relationships and elucidate mechanisms underlying medication combination effects on patient‐specific characteristics, thereby enabling personalized medication regimen recommendations. Our research highlights the decomposition of complex clinical problems, analytical problem‐solving workflows, and the integration of real‐world clinical data challenges, including data quality issues and confounding factors. By synergizing CoD, deep learning architectures, and intelligent decision‐making methods incorporating expert experience metrics, we propose a “data‐first, problem‐driven” research framework. The deep learning model under CoD paradigm explicitly clarifies relationships between medication strategies and clinical diagnostic information, enhancing interpretability of clinical diagnosis and intervention outcomes while providing clinicians with transparent, trustworthy decision support.

The required data for model implementation can be sourced from electronic health records, medical insurance databases, and patient registry systems, enabling rapid collection of large‐scale, diverse clinical cases including diagnostic, therapeutic processed, and clinical outcomes. This approach facilitates observation of comprehensive intervention effects (e.g., medication combinations, and therapeutic sequencing) in real‐world clinical settings rather than single‐factor controlled environments, ensuring better alignment with actual clinical practice and improved translational potential. Future enhancements to MDD‐CoD will focus on integrating microscopic multimodal pre‐trained models into patient‐medication personalization workflows and developing single‐modality therapeutic recommendation methods. These advancements aim to improve the framework's precision, applicability across diverse CDs categories (e.g., respiratory, cardiovascular, and metabolic disorders), and capacity to mitigate overdiagnosis and overtreatment challenges in clinical practice.

## 6 Experimental Section

### Datasets

This study is a multi‐center, retrospective research where clinical data from four CDs were utilized from Guangdong Provincial Hospital of Chinese Medicine, Jinan University, and Zhongshan Hospital of Traditional Chinese Medicine to train and validate the model. Through the data preprocessing procedure summarized in Appendix 1, a dataset of personalized medication for CDs was constructed, and ultimately, 181 patients with CKD, 161 patients with RA, 3173 patients with KOA, and 160 patients with CRC were included. Since MN is the most common cause of nephrotic syndrome in adults and has a large sample size (72 cases) in the CKD dataset, it was independently evaluated as a disease label for subsequent experiments and analyses. Thus, this study constructed five datasets covering four CDs. The multimodal data for CKD and MN included high‐resolution pathological section images and laboratory indicators. The multimodal data for RA comprised joint imaging and laboratory indicators, while the multimodal data for CRC included local low‐resolution pathological section images and laboratory indicators. For KOA, only laboratory indicators were available. Additionally, medication information encompassed 901 CKD medication regimens and 325 individual medications (both herbal medicines​​ and conventional drugs); MN had 186 medication regimens and 211 individual medications; RA had 1101 medication regimens and 358 individual medications. The baseline characteristics of all participants are summarized in Tables S1 to S5 of Appendix 1.

### Statistical Analysis

All data were analyzed statistically using IBM SPSS Statistics 26.0. Continuous variables underwent normality testing via the Shapiro‐Wilk test. After normality testing for age variables, if they followed a normal distribution, grouping was based on mean±SD; if not normally distributed, grouping was based on quartiles. Categorical variables were expressed as frequencies and percentages. For comparisons between groups, categorical variables were analyzed using Pearson's chi‐square test; when data did not meet the conditions for applying the chi‐square test, Fisher's exact test was used. All tests were two‐sided, and a *p* <0.05 was considered statistically significant.

### Data Preprocessing

This study implemented rigorous quality control and standardization procedures for multimodal data. For image data, tissue foreground regions were extracted using threshold segmentation, and blank background slices were excluded. For consecutive sections and color Doppler ultrasound images from the same patient, only diagnostically valuable images were retained. For tabular data, missing values were imputed with the median of the corresponding field (confirmed to follow a non‐normal distribution), and outliers exceeding the physiological range thresholds defined by clinical guidelines were removed. For medication regimen data, medication names were standardized, duplicate treatment plans and medications not relevant to the treatment of the disease were excluded. For protein network data related to medications, self‐loops and duplicate edges were removed (with edge weights set to the maximum value). For datasets with significant class imbalance, “RandomOverSampler” was applied in the “development set”, while the test set retained its original distribution to evaluate real‐world performance.

### Architecture of the MDD‐CoD Framework

**Figure 5:**
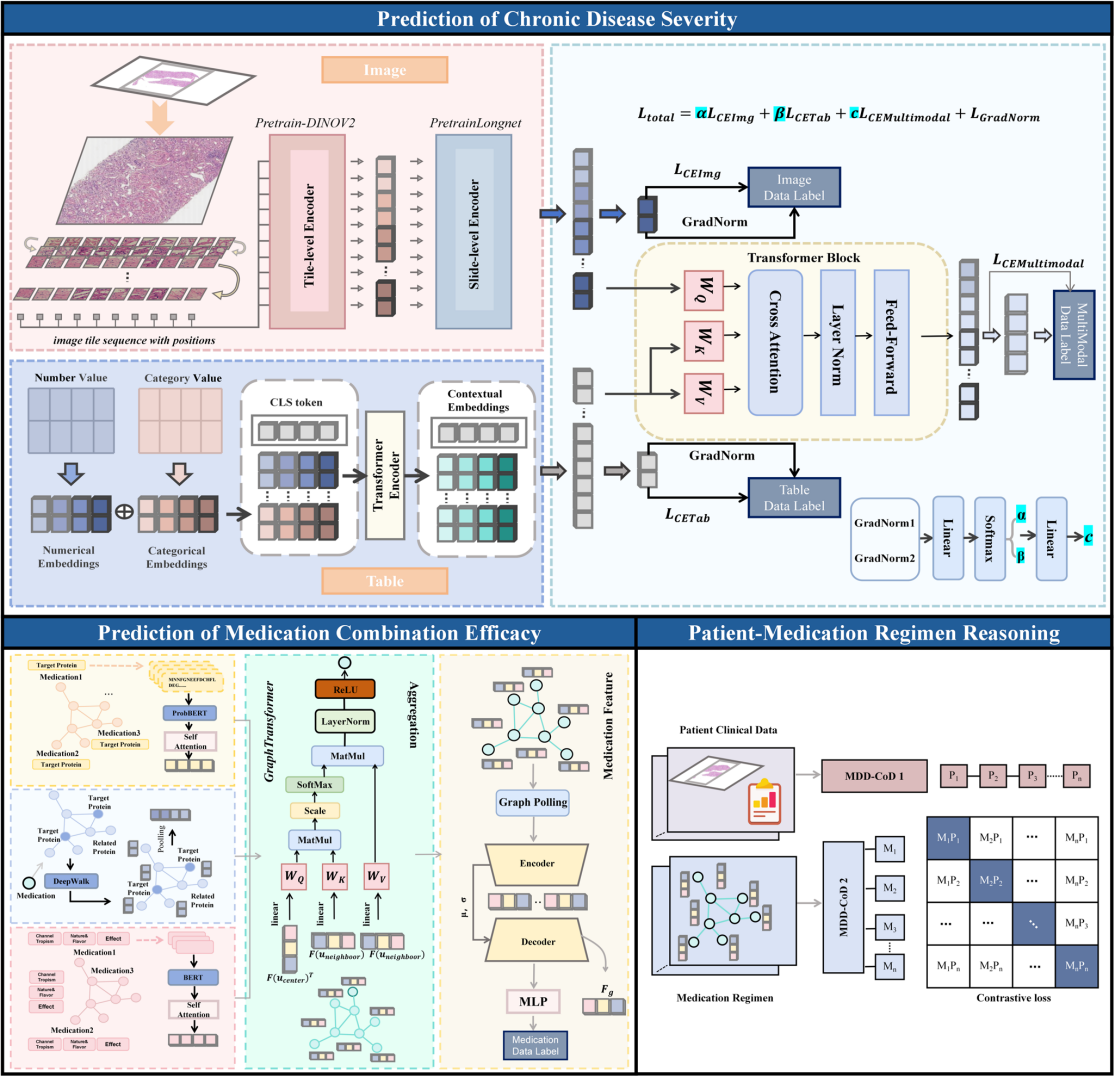
Architecture of the MDD‐CoD framework.

We propose a MDD‐CoD framework, where three‐stage deep learning tasks (MDD‐CoD1, MDD‐CoD2, and MDD‐CoD3) are sequentially organized to reflect upstream–downstream logical dependencies, thereby forming a coherent clinical decision‐making process. The overview of the MDD‐CoD framework is shown in **Figure** **5**, details provided in Appendix 2.

### MDD‐CoD1

MDD‐CoD1 primarily addresses multimodal feature embedding, alignment, and fusion tasks involving histopathological and joint ultrasound images, as well as tabular data from laboratory indicators. Specifically, distinct feature embedding strategies are applied to different modalities. For histopathological images, feature embedding is implemented using pre‐trained Giga‐provpath^[^ ^61^ ^]^ and HIPT frameworks.^[^ ^62^ ^]^ For ultrasound images, features are extracted via a pre‐trained Vision Transformer (ViT) model. Additionally, for patients with multiple images, an averaging operation is applied to the features of each image. The resultant image features are denoted as *E*_Image_. For tabular data encompassing numerical and categorical laboratory indicators, we leverage the FT‐Transformer model,^[^ ^63^ ^]^ capitalizing on its robust capability for tabular data processing. This is achieved through two core components: the Feature Tokenizer and the TransformerBlock. First, numerical and categorical features in the tabular data are converted into embedding vectors, represented as $T_{n}^{\left(\mathrm{num}\right)},T_{m}^{\left(\mathrm{cat}\right)}$, where n and m denote the number of numerical and categorical fields, respectively. These embeddings are then concatenated (stacked) to form a comprehensive tabular feature representation $T=\mathrm{stack}\left[T_{1}^{\left(\mathrm{num}\right)},\text{…},T_{n}^{\left(\mathrm{num}\right)},T_{1}^{\left(\mathrm{cat}\right)},\text{…},T_{m}^{\left(\mathrm{cat}\right)}\right],\;T\in R^{(n+m)\times d}$. Finally, the concatenated features are fed into the TransformerBlock to generate CLS features—global representations that encapsulate cross‐field interactions and fusion—denoted as *E*_Table_.

Following the completion of feature embedding across all modalities, we perform multimodal alignment and fusion. A cross‐attention mechanism is employed to establish inter‐modal interaction relationships:

(1) $$\begin{array}{c} E_{\mathrm{patient}}=\mathrm{Softmax}\left(\frac{E_{Table}W_{Q}{\left(E_{Image}W_{K}\right)}^{⊤}}{\sqrt{d}}\right)E_{Image}W_{V}, \end{array}$$

where *W*_*q*_, *W*_*k*_, and *W*_*v*_ are learnable weight matrices. In this process, the tabular feature *E*_Table_ is mapped to a query vector via *W*_*q*_, while the image feature *E*_Image_ is projected into a key vector through *W*_*k*_. The similarity matching between these vectors generates an attention score matrix, which is then aggregated with *W*_*v*_ to produce fused cross‐modal patient features. Layer normalization and residual connections are applied to maintain computational stability. The fused cross‐modal patient features, denoted as *E*_*Fusion*_, are fed into a classification layer for downstream task predictions.

Gradient norms are employed to quantify the predictive contributions of the image modality *E*_Image_ and tabular modality *E*_Table_ to disease stratification. During model training, these gradient norms are formulated as:

(2) $$\begin{array}{c} {\left\|\nabla L_{\mathrm{Image}}\right\|}_{2}=\sqrt{\sum_{1}{\left(\frac{\partial L_{\mathrm{Image}}}{\partial {\tilde{\Theta }}_{1}}\right)}^{2}}\mathrm{and}{\left\|\nabla L_{\mathrm{Table}}\right\|}_{2}=\sqrt{\sum_{1}{\left(\frac{\partial L_{\mathrm{Table}}}{\partial {\tilde{\Theta }}_{1}}\right)}^{2}}, \end{array}$$

where θ_*i*_ denotes the trainable parameters of the model, ‖ · ‖_2_ represents the L2 norm, and $L_{\mathrm{Image}}$, $L_{\mathrm{Table}}$ correspond to the loss functions for the image and tabular modalities, respectively. The combined gradient regularization term $L_{\mathrm{GradNorm}}$ is defined as:

(3) $$L_{\mathrm{GradNorm}}=∥\nabla L_{\mathrm{Image}}{∥}_{2}+∥\nabla L_{\mathrm{Table}}{∥}_{2}.$$

To ensure stable estimation of modality contributions, these gradient statistics are fed into a three‐layer nonlinear projection network. The second layer employs a Sigmoid activation function to guarantee non‐negativity of output weights, while the third layer utilizes softmax for normalized weight allocation, thereby generating weighted contribution weights for the image and tabular modalities, denoted as *w*_*Image*_ and *w*_*Table*_, respectively.

The contribution weights of both modalities are subsequently fed into a two‐layer nonlinear projection network to derive the modality fusion weight, denoted as *w*_*Fusion*_, where the second layer employs a softplus activation function. The total loss function incorporates both unimodal and multimodal classification losses, formulated as:

(4) $$\begin{array}{ccc} L_{\mathrm{fusion}} & = & w_{\mathrm{Image}}\cdot L_{\mathrm{Image}}+w_{\mathrm{Table}}\cdot L_{\mathrm{Table}}+\\ & & w_{\mathrm{Fusion}}\cdot L_{\mathrm{Multimodal}}+L_{\mathrm{GradNorm}}. \end{array}$$

### MDD‐CoD2

MDD‐CoD2 mainly considers the multiple properties (Properties, Protein‐Seq, PPI) of a medication regimen to mine the interactions between medications within the medication regimen, which is achieved by constructing a medication graph model.

Firstly, a medication regimen is defined as a graph structure denoted as *G* = (*V*, *E*), where V represents the medication nodes within the graph and E represents the connectivity of the medication nodes within the graph. The initial values of the edges are represented by a priori knowledge graph consisting of medication linkage relationships. The node features are stitched together from multiattribute medication features and are computed as:

(5) $$\begin{array}{c} h_{i}=\mathrm{Concat}\left(F_{\mathrm{macro},i},F_{\mathrm{micro},i},F_{\mathrm{ppi},i}\right)\in R^{D_{\mathrm{node}}}, \end{array}$$

where F_*macro*_, F_*micro*_, and F_*ppi*_ denote the feature embedding of Properties, Protein‐Seq and PPI, respectively.

Secondly, the medication multi‐property Properties, Protein‐Seq and PPI are subjected to feature extraction through Bert‐Chinese and ProbBERT pre‐trained language models, fusion with self‐attention mechanism, and Deepwalk model, respectively. In particular, for the feature Text_*i*_ of the property Properties, it is extracted by Bert‐Chinese as:

(6) $$\begin{array}{ccc} T_{i} & = & {\mathrm{BERT}}_{\mathrm{Chinese}}\left({\mathrm{Text}}_{i}\right)\;\forall i\\ & & \in \{\mathrm{Meridian},\mathrm{Property},\mathrm{Flavours},\mathrm{Side}\mathrm{Effects}\}, \end{array}$$

which is then fed into the attention mechanism model to derive the embedded feature of the property Properties: $F_{\mathrm{macro}}=\mathrm{Attention}\left(Q_{T},K_{T},V_{T}\right)$.

The attribute PPI is a network *M* = (*V*, *E*), and feature embedding is performed on network *M* using Deepwalk, which contains a random walk with a Word2Vec component. Specifically, a random walk is performed from the initial node at step 1 to generate a random walk sequence within the PPI. *v*_*k*_ represents the current protein node, and *N*(*v*_*k*_) denotes the neighbors of node *v*_*k*_. A node *v*_*k*_+1 is randomly selected from the set of neighbors of the current node *v*_*k*_, **generating a walk sequence of length l**, denoted as: Walk = [*v*_1_, *v*_2_, …, *v*_*l*_] , where $\;v_{k+1}∼\mathrm{Uniform}\left(N\left(v_{k}\right)\right)$, the random wandering sequences within the PPI are then fed into the Word2Vec model for training. By maximizing the co‐occurrence probability of the target node within the PPI with its contextual neighboring nodes, vector representations of nodes with similar network neighborhood structures that are similar in the embedding space are obtained. In addition, the formula for calculating the weights of edges between nodes is denoted as: $w_{ij}=\frac{co-occurrence(i,j)}{\max_{\left(i^{\prime },j^{\prime }\right)}co-occurrence\left(i^{\prime },j^{\prime }\right)}$, the *co* − *occurrence*(*i*′, *j*′) refers to the number of co‐occurrences of the target medication nodes, and $\max_{\left(i^{\prime },j^{\prime }\right)}co-occurrence\left(i^{\prime },j^{\prime }\right)$ is the number of all co‐occurrences of all medications.

Once the feature embedding tasks for the three attributes are completed, the construction of the medication regimen graph is formed. Next, the graph structure data is trained using two GNN‐Transformer convolutional layers.^[^ ^64^ ^]^ In this process, the GNN‐Transformer updates the representation of nodes by modeling the similarity between medication nodes. Specifically, the core of GT is an attention mechanism of each neighbor. In addition, in the graph aggregation operation in each layer, the edge weights are added to the key. This determines the intensity of information dissemination, denoted as α_*ij*_. It is calculated as:

(7) $$\begin{array}{cc} \alpha_{ij} & =\frac{\exp\left(\frac{h_{i}W_{Q}{\left(h_{j}W_{K}+w_{ij}\right)}^{⊤}}{\sqrt{d_{k}}}\right)}{\sum_{k\in N(i)}\exp\left(\frac{h_{i}W_{Q}{\left(h_{k}W_{K}+w_{ik}\right)}^{⊤}}{\sqrt{d_{k}}}\right)}, \end{array}$$

(8) $$\begin{array}{cc} h_{i}^{\prime } & =\sigma \left(\sum_{j\in N(i)}\alpha_{ij}h_{j}W_{V}\right)\;, \end{array}$$

*N*(*i*) is the node of the i‐th neighbor, *h*_*i*_ is the target node feature representation, *h*_*i*_ is the neighbor node feature, and *h*_*k*_ is the collective name of all the neighbor features. σ(·) denotes the activation function. $W_{Q}\in R^{d\times d_{q}}$, $W_{K}\in R^{d\times d_{k}}$ and $W_{V}\in R^{d\times d_{v}}$ are learnable weight matrices for qureys, keys and values respectively.

To align the specificity of different medication attribute features, the cross‐scale feature alignment method of variational autoencoder (VAE) is employed. Specifically, the multi‐scale medication regimen features are mapped to Gaussian distributed hidden variables by the encoder, and after reparameterized sampling, the features are reconstructed by the decoder to achieve cross‐modal alignment and update of the medication node representations. The output is the medication regimen feature denoted as *E*_medication_, and the alignment loss is calculated as follows:

(9) $$\begin{array}{c} L_{\mathrm{KL}}=\mathrm{KL}\left(q\left(z|h_{i}^{\left(2\right)}\right)∥p\left(z\right)\right)=\frac{1}{2}\sum \left(\mu_{i}^{2}+\sigma_{i}^{2}-\log\sigma_{i}^{2}-1\right), \end{array}$$

where $q(z|h_{i}^{\left(2\right)})$ is the variational posterior, *p*(**z**) is the prior and µ_*i*_, σ_*i*_ are learned mean and std for node *i*.

In addition, to mitigate the category imbalance, we use Focal Loss,^[^ ^65^ ^]^ calculated as: $L_{\mathrm{focal}}=-\alpha_{t}{\left(1-p_{t}\right)}^{\gamma }\log\left(p_{t}\right)$, where *p* is the predicted probability that the sample belongs to the correct category, and γ is the focusing parameter whose value can be adjusted to make the model focus more on difficult samples. The final loss function is calculated as: $L_{\mathrm{total}}=L_{\mathrm{KL}}+L_{\mathrm{focal}}$.

### MDD‐CoD3

MDD‐CoD3 employs contrast learning to reason about the alignment and matching of patient features and medications, including the tasks of feature dimension alignment using MLP after obtaining patient features and medication regimen features, and matching based on contrast loss. Specifically, the formula for calculating the contrast loss between patient features and medication regimen features is as follows:

(10) $$\begin{array}{ccc} L_{X\to Y} & = & \frac{1}{2N}\sum_{i=1}^{N}{\left[\left(s\left(E_{X,i},E_{Y,i}^{+}\right)\right)-\left(s\left(E_{X,i},E_{Y,i}^{-}\right)\right)+\alpha \right]}_{+}\\ & & \;(X,Y\in \{\mathrm{medication},\mathrm{patient}\}), \end{array}$$

where *s* is the cosine similarity function, $E_{Y,i}^{+}$/$E_{Y,i}^{-}$ are positive/negative samples, α is the hyperparameter enforcing separation strength.

The total loss is the average of the losses in both directions (medication regimen to patient and patient to medication regimen), ensuring that the distances between the medication and the clinical embedding converge in semantic space. The total loss is calculated as follows:

(11) $$\begin{array}{c} L_{\mathrm{total}}=\frac{1}{2}\left(L_{\mathrm{medication}\to \mathrm{patient}}+L_{\mathrm{patient}\to \mathrm{medication}}\right). \end{array}$$

Ultimately, the patient‐medication regimen matching confidence will be expressed as:

(12) $$\begin{array}{c} \mathrm{Confidence}\left(E_{\mathrm{patient}},E_{\mathrm{medication}}\right)=\frac{E_{\mathrm{patient}}\cdot E_{\mathrm{medication}}}{\left\|E_{\mathrm{patient}}\right\|\left\|E_{\mathrm{medication}}\right\|}. \end{array}$$

## Conflict of Interest

The authors declare no conflict of interest.

## Supporting information

Supporting Information

## Data Availability

The data that support the findings of this study are available on request from the corresponding author. The data are not publicly available due to privacy or ethical restrictions.
