# Supplementary material for: Personalized Medication for Chronic Diseases Using Multimodal Data‐Driven Chain‐of‐Decisions
Source: Adv Sci (Weinh). 2025 Aug 11;12(40):e04079. doi: 10.1002/advs.202504079 (PMC12561204; doi:10.1002/advs.202504079)
Supplement: Supplementary file 1 — Supporting Information [file ADVS-12-e04079-s001.pdf]

Supporting Information

Personalized Medication for Chronic Diseases using Multimodal Data-Driven Chain-of-Decisions

*Xiaoli Chu [1, †], Yiheng Ye [2, †], Siqiao Tang [3, †], Miaoru Han [3, †], Guowei Wang [4, †], Shuai Lin [5, †], Bingzhen Sun [6], Qingchun Huang [7], Yan Zhang [8, *], Xiaodong Chu [9, *], Kun bao [10, *]*

[1] State Key Laboratory of Traditional Chinese Medicine Syndrome / Department of TCM Big Data Research, The 2nd Affiliated Hospital of Guangzhou University of Chinese Medicine, Guangdong, China;

[2] School of Information Science, Guangdong University of Finance & Economics, Guangdong, China;

[3] State Key Laboratory of Dampness Syndrome of Chinese Medicine, The 2nd Affiliated Hospital of Guangzhou University of Chinese Medicine, Guangdong, China;

[4] School of Electronic and Information Engineering, South China University of Technology, Guangdong, China;

[5] Department of Nephrology, Zhongshan Hospital of Traditional Chinese Medicine Affiliated to Guangzhou University of Chinese Medicine,Guangdong, China;

[6] School of Economics and Management, Xidian University, Xi'an, China;

[7] Department of Rheumatology, The 2nd Affiliated Hospital of Guangzhou University of Chinese Medicine, Guangdong, China;

[8] Research Center of Intelligent Computing and Big Data Technology / School of Digital Economics, Guangdong University of Finance & Economics, Guangdong, China;

[9] Cancer Research Institute / College of Pharmacy / The 1st Affiliated Hospital, Jinan University, Guangdong, China;

[10] State Key Laboratory of Dampness Syndrome of Chinese Medicine / Department of Nephrology, The 2nd Affiliated Hospital of Guangzhou University of Chinese Medicine, Guangdong, China

†These authors contributed equally to this work.

TABLE OF CONTENTS

Appendix 1：Datasets.......... 3
Appendix 2：MDD-CoD Model Architecture Diagram.......... 8
Appendix 3：Training Details.......... 12
Appendix 4：Evaluation of Indicators.......... 14
Appendix 5：Comparative Advantages of Core Technologies in MDD-CoD.......... 16
Appendix 6：Model Generalizability Between Diseases and Hospitals.......... 18
Appendix 7：MDD-CoD1 Interpretability Study.......... 20
Appendix 8：Abbreviation of Medication.......... 21

Appendix 1：Datasets

Regarding the generalizability of the model between diseases and hospitals. The complete multimodal datasets for these diseases (CKD, MN, RA, CRC,) are rare datasets, and the collection of clinical characteristics and pathological mechanisms for each disease requires different devices and processes, making the collection and analysis of relevant data challenging. To the best of our knowledge, there currently exists no publicly available dataset worldwide that simultaneously incorporates multimodal clinical data and Chinese and Western medicine data for the chronic diseases at a comparable scale. The dataset we have constructed (CKD, MN, RA, CRC) fills this gap, aiding researchers in conducting rare disease research.

The inclusion and exclusion criteria for data collection are as follows: Inclusion criteria: ① Patients with a clear diagnosis and complete medical records of Chronic Kidney Disease (CKD), Rheumatoid Arthritis (RA), Colorectal Cancer (CRC); or Knee Osteoarthritis (KOA). ② The dataset must include at least one type of structured laboratory test or imaging information, as well as medication information. Exclusion criteria: ① Severe missing data or insufficient follow-up data; ② Patients with other major systemic comorbidities.

A renal biopsy of histopathological slices was performed by nephrologists from Guangdong Provincial Hospital of Traditional Chinese Medicine and Zhongshan City Hospital of Traditional Chinese Medicine for a renal puncture biopsy. Histopathological slices of renal biopsies were selected under PAS stain. Colorectal biopsy histopathological slides were taken by a specialist from Jinan University who performed a gross biopsy of the excised tissue and selected colorectal histopathological slides under hematoxylin-eosin (HE) staining. Hamamatsu's pathology digital slide scanner was used to capture the whole slide imaging (WSIs) of the sample slides, and all the contaminated and poorly stained slides were excluded and saved as '.ndpi' data format files. Rheumatoid arthritis joint ultrasound images were examined by ultrasound specialists of Guangdong Provincial Hospital of Traditional Chinese Medicine (GDHTC) and saved as DCM files, and Sante DICOM

Viewer Lite (v4.0.2) was used to export each image in the DCM files to a '.jpg' format file.

Table S1. Baseline characteristics of RA dataset

Clinical Feature	Number of Cases	Disease Activity Score in 28 Joints				χ^2	*P*
		Remission	Low Disease Activity	Moderate Disease Activity	High Disease Activty		
**Age**						10.109	0.271
<50	43(26.7)	5(11.6)	1(2.3)	15(34.9)	22(51.2)		
50~58	43(26.7)	3(7.0)	1(2.3)	15(34.9)	24(55.8)		
58~67	36(22.4)	1(2.8)	1(2.8)	11(30.5)	23(63.9)		
>67	39(24.2)	0(0)	1(2.6)	8(20.5)	30(76.9)		
**Gender**						1.601	0.65
Male	22(13.7)	1(4.5)	1(4.5)	8(36.5)	12(54.5)		
Female	139(86.3)	8(5.8)	3(2.2)	41(29.5)	87(62.6)		
						5.43	0.796
0	38(23.6)	1(2.6)	1(2.6)	12(31.6)	24(63.2)		
1	63(39.1)	3(4.8)	1(1.6)	19(30.2)	40(63.5)		
2	45(28.0)	4(8.9)	2(4.4)	11(24.4)	28(62.2)		
3	15(9.3)	1(6.6)	0(0)	7(46.7)	7(46.7)		
**Synovial blood flow**						4.735	0.881
0	45(28.0)	3(6.7)	1(2.2)	12(26.7)	29(64.4)		
1	53(32.9)	3(5.7)	1(1.9)	13(24.5)	36(67.9)		
2	43(26.7)	2(4.7)	2(4.7)	16(37.2)	23(53.5)		
3	20(12.4)	1(5.0)	0(0)	8(40.0)	11(55.0)		
**Bone erosion**						1.23	0.76
Yes	140(87.0)	9(6.4)	4(2.9)	43(30.7)	84(60.0)		
No	21(13.0)	0(0)	0(0)	6(28.6)	15(71.4)		

As show in Table S1, age was non-normally distributed according to the Shapiro–Wilk test and was categorized based on quartiles (Q1=50, Q2=58, Q3=67 years). Group comparisons were performed using Pearson's chi-square test. Two-sided *P* values < 0.05 were considered statistically significant.

Table S2. Baseline characteristics of CKD dataset

Clinical Feature	Number of Cases	CKD Stage			χ^2	*P*
		1	2	3~5		
**Age**					9.07	0.059

<30	26(23.9)	20(76.9)	2(7.7)	4(15.4)		
30~50	61(56.0)	37(60.7)	16(26.2)	8(13.1)		
≥50	22(20.1)	9(40.9)	6(27.3)	7(31.8)		
**Gender**					0.051	0.975
Male	57(52.3)	34(59.7)	13(22.8)	10(17.5)		
Female	52(47.7)	32(61.5)	11(21.2)	9(17.3)		
**Pathological Lesion**					31.691	0.000
Mild Lesion	48(44.0)	41(85.4)	6(12.5)	1(2.1)		
Moderate Lesion	48(44.0)	23(47.9)	14(29.2)	11(22.9)		
Severe Lesion	13(12.0)	2(15.4)	4(30.8)	7(53.8)		

As show in Table S2, age followed a normal distribution as assessed by the Shapiro–Wilk test (P = 0.107). The mean ± standard deviation of age was 40.83 ± 12.55 years. Age was categorized based on clinically relevant cutoffs into three groups: young (<30 years), middle-aged (30–50 years), and elderly (≥50 years). Pathological lesion was assessed according to Katafuchi Score.

Table S3. Baseline characteristics of MN dataset

Clinical Feature	Number of Cases	Ehrenreich-Churg Stage			χ^2	*P*
		**I**	**II**	**III**		
**Age**					3.035	0.899
<34	20(27.8)	1(5.0)	18(90.0)	1(5.0)		
34~50	16(22.2)	1(6.3)	15(93.8)	0(0)		
50~60	19(26.4)	3(15.8)	15(78.9)	1(5.3)		
>60	17(23.6)	2(11.8)	14(82.4)	1(5.9)		
**Gender**					0.799	0.851
Male	49(68.1)	4(8.2)	43(87.8)	2(4.1)		
Female	23(31.9)	3(13)	19(82.6)	1(4.3)		
**Risk Assessment**					1.921	0.726
Low Risk	5(6.9)	1(20.0)	4(80.0)	0(0)		
Moderate Risk	28(38.9)	3(10.7)	24(85.7)	1(3.6)		
High Risk	39(54.2)	3(7.7)	34(87.2)	2(5.1)		

As show in Table S3, age was non-normally distributed according to the Shapiro–Wilk test and was categorized based on quartiles (Q1=34, Q2=49.5, Q3=60 years). Group comparisons were performed using Pearson's chi-square test. Two-sided *P* values < 0.05 were considered statistically significant. Ehrenreich–Churg staging was used for membranous nephropathy classification based on electron microscopy: Stage I = small subepithelial electron-dense deposits without GBM reaction; Stage II = deposits surrounded by projections of GBM forming "spikes" or new basement membrane material; Stage III = deposits incorporated within thickened

and irregular GBM; Stage IV (not included here) = resolution of deposits with persistent GBM alterations. Risk categories (low, moderate, and high) were determined based on clinical criteria outlined in the 2021 KDIGO guideline for glomerular diseases, considering parameters such as eGFR, proteinuria, serum albumin, and anti-PLA2R antibody levels.

Table S4. Baseline characteristics of CRC dataset

Clinical Feature	Number of Cases	Degree of stenosis		χ^2	P
		<50%	>50%		
**Age**				2.238	0.536
<57	43(26.9)	27(62.8)	16(37.2)		
57~69	41(25.6)	23(56.1)	18(43.9)		
69~78	41(25.6)	26(63.4)	15(36.6)		
>78	35(21.9)	17(48.6)	18(51.4)		
**Gender**				1.003	0.317
Male	105(65.6)	64(61.0)	41(39.0)		
Female	55(34.4)	29(52.7)	26(47.3)		
**TNM Stage**				5.375	0.068
I	44(27.5)	32(72.7)	12(27.3)		
II	98(61.2)	52(53.1)	46(46.9)		
III	18(11.3)	9(50.0)	9(50.0)		
Degree of differentiation				8.451	0.038
Well differentiated	24(15.0)	20(83.3)	4(16.7)		
Moderately differentiated	62(38.8)	31(50.0)	31(50.0)		
Poorly differentiated	56(35.0)	33(58.9)	23(41.1)		
Undifferentiated	18(11.2)	9(50.0)	9(50.0)		
**Survival Time**				1.496	0.221
Alive	49(30.6)	32(65.3)	17(34.7)		
Deceased	111(69.4)	61(55.0)	50(45.0)		

As show in Table S1, age was non-normally distributed according to the Shapiro–Wilk test and was categorized based on quartiles (Q1=57, Q2=69, Q3=78 years). Group comparisons were performed using Pearson’s chi-square test. Two-sided *P* values < 0.05 were considered statistically significant. “*Degree of stenosis*” was determined based on the estimated luminal narrowing rate: <50% indicates mild to moderate stenosis, and >50% indicates severe stenosis. TNM stage (Tumor–Node–Metastasis classification) was determined according to the 8th edition AJCC criteria: Stage I (T1–T2, N0, M0), Stage II (T3–T4, N0, M0), and Stage III (any T, N1–2, M0); Stage 0 and IV were not included in this dataset. Tumor

differentiation was graded histologically as well, moderately, poorly, or undifferentiated, based on the degree to which tumor cells resemble normal glandular epithelium.

Table S5. Baseline characteristics of KO dataset

Clinical Feature	Number of Cases	Kellgren-Lawrence grading system				χ^2	P
		0	I	II	III		
**Age**						12.897	0.167
<60	794(25.0)	197(24.8)	315(39.7)	250(31.5)	32(4.0)		
60~67	895(28.2)	185(20.7)	375(41.9)	278(31.1)	57(6.4)		
67~74	714(22.5)	177(24.8)	295(41.3)	204(28.6)	38(5.3)		
>74	770(24.3)	176(22.9)	305(39.6)	236(30.6)	53(6.9)		
**Gender**						5.876	0.118
Male	849(26.8)	218(25.7)	338(39.8)	254(29.9)	39(4.6)		
Female	2324(73.2)	517(22.2)	952(41)	714(30.7)	141(6.1)		

As show in Table S5, age was non-normally distributed according to the Shapiro–Wilk test and was categorized based on quartiles (Q1=60, Q2=67, Q3=74 years). Group comparisons were performed using Pearson's chi-square test. Two-sided P values < 0.05 were considered statistically significant. The Kellgren–Lawrence grading system was used to assess radiographic severity of osteoarthritis: grade 0 = no radiographic features of OA, grade I = doubtful narrowing of joint space and possible osteophytic lipping, grade II = definite osteophytes and possible joint space narrowing, grade III = multiple osteophytes, definite narrowing of joint space, and possible deformity of bone ends.

Appendix 2：MDD-CoD Model Architecture Diagram

The implementation code for the model proposed in this study has been open-sourced on GitHub and is publicly available. The repository URL is: https://github.com/yhengYE/MDD-CoD. All configuration details, preprocessing scripts, and model architecture definitions required for complete experiment reproduction are included in this repository. The complete dataset is subject to privacy and ethical restrictions (IRB: BE2025-098-01, BE2024-325-01, 2024ZSZY-LLK-408, and JNUKY-2023-0105) and cannot be publicly shared. To obtain the dataset, please contact the corresponding author and sign the relevant confidentiality agreement before accessing it.

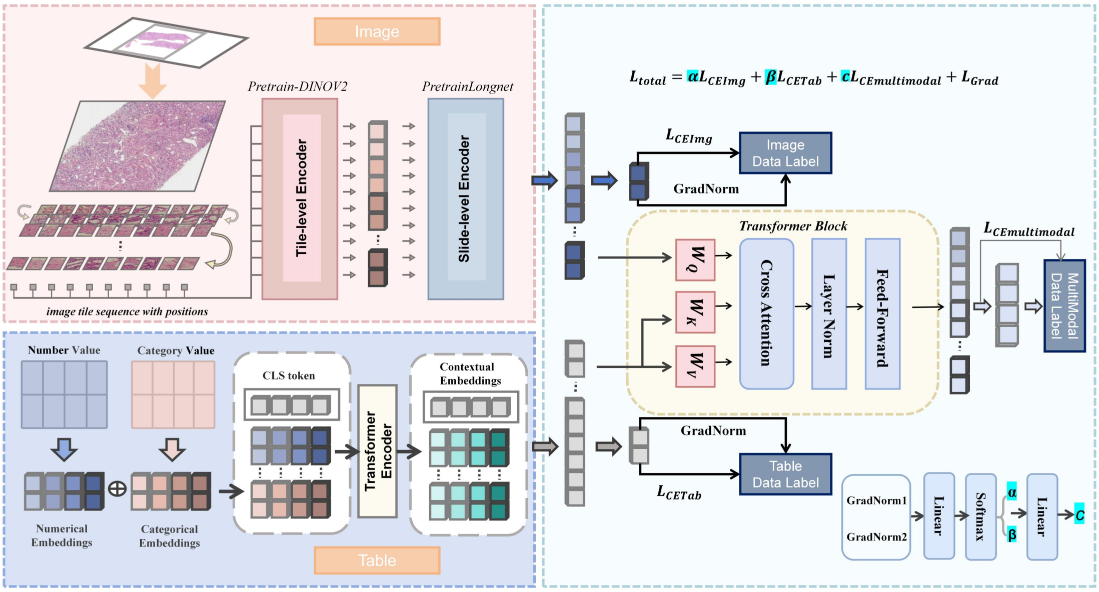

Figure S1. MDD-CoD1： The multi-modal feature embedding and multi-task prediction model based on Transformer improves the definition of disease classification.

As shown in Figure S1, a multi-modal fusion framework based on the multi-task thought is proposed, which regards the feature representation process of each mode as a task, and the alignment and fusion of multi-modal features as a global task. This framework can be applied to downstream tasks that address the weight of different modes in clinical decision making. Specifically, each single mode carries out feature learning through its own pre-trained feature extractor, and then carries out information

exchange between modes through the cross-attention mechanism introduced by the global task. Finally, the common loss of single mode and multi-mode is synthesized, the loss optimization of the overall model is carried out, and comprehensive clinical decision is obtained, and the proportion of different clinical modes in decision making is obtained.

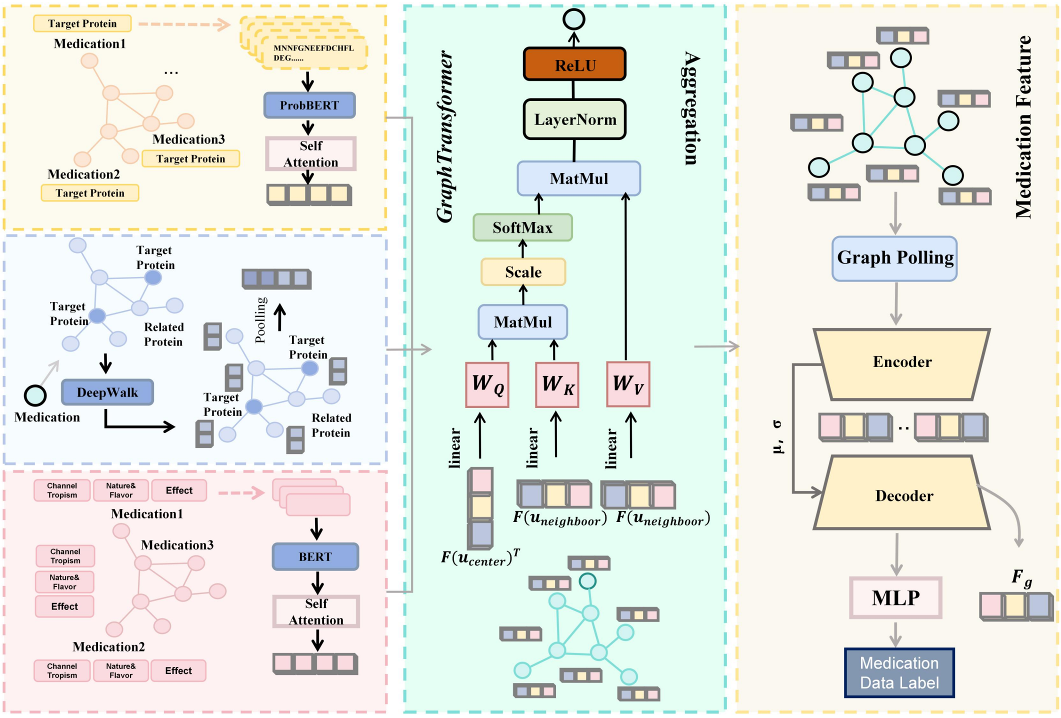

Figure S2. MDD-CoD2: Graph transformer network-based drug marco-micro information fusion model to analyze the therapeutic mechanism of drug combination.

As shown in Figure S2, a graph neural network based on the three modalities of drug information is constructed. On the one hand, for macro pharmacological modal information, BERT-Chinese pre-training model is used to embed text features. In addition, a fusion module based on attention mechanism is introduced to merge pharmacological information of multiple dimensions to obtain a unified feature representation. This feature can represent the macro information of the drug. On the other hand, for microscopic modal information, ProbBERT pre-training model is used to extract drug target characteristics. The pre-training model has been pre-trained on large-scale protein sequence data sets, and can better understand the meaning of protein amino acid sequence and give high-quality feature representation. Then we enter the attentional mechanism and fuse multiple protein sequences of a single drug

to obtain a uniform representation of protein characteristics. This feature can represent the microscopic protein information of the drug. In addition, the micro-characterization also incorporates a protein-protein interaction network (PPI) of the drug target based on DeepWalk, aiming to build a comprehensive representation of the PPI characteristics.

Macro and micro drug prescription information features (including macro drug, micro protein properties, and micro protein network structure) are prepared for splicing operation, and a unified macro and micro drug prescription feature representation was obtained. Then, a drug prescription is regarded as a subnetwork, a drug is a network node, and macro and micro drug prescription features are node features. The Gnn-Transformer model is used to reconstruct and aggregate the network to obtain the macro and micro representation of drug prescription network after fusion based on GNN. After the pooling operation, the macro and micro drug prescription network obtains the prescription feature representation, which is sent to the VAE module for the alignment of multi-modal data. The input features are compressed into a hidden space representation, and the macro and micro drug prescription feature representations are reconstructed.

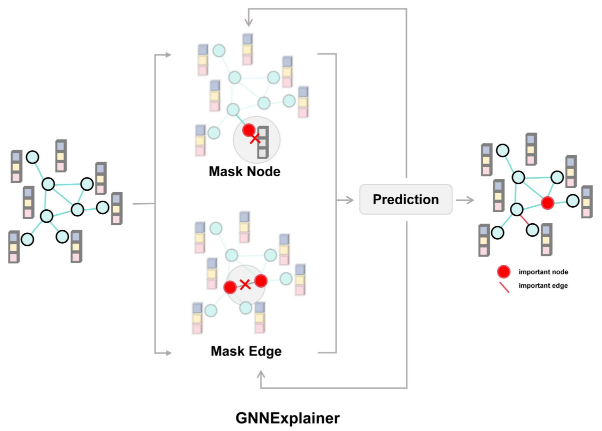

Figure S3. Interpretability of the MDD-CoD2 model.

As shown in Figure S3, Graph neural network interpretable tool GNN-explainer is used to get the clinical hints in Chain2. GNN-explainer, through a trainable masking mechanism, dynamically identifies the most critical substructures of the network for a specific prediction. Specifically, it assigns importance scores to the

node and edge features in the drug network and, through an optimization process, finds a concise subgraph that can reproduce the original prediction results to the greatest extent in the GNN model.

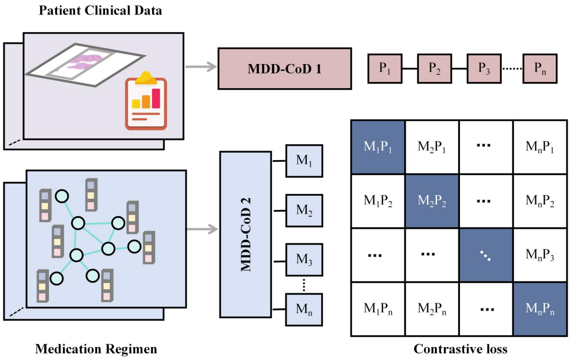

Figure S4. MDD-CoD3：A cross-modal shared space representation model based on contrast learning for disease and medication regimen matching.

The above two chain prepare the patient's multi-modal clinical features and macro-micro medication prescription features, and the stage3 (as shown in Figure S4) builds CLIP model based on these two types of features. Specifically, multimodal clinical features and macro- micro drug prescription features are preprocessed and fed into the two inputs of the CLIP model, respectively, to embed into a shared semantic space for constructing positive and negative sample pairs. Then, through the InfoNCE loss optimization model of comparative learning, clinical features and prescription features are effectively aligned in semantic space, and accurate correlation modeling of the two types of features is realized.

Appendix 3 ：Training Details

MDD-CoD1: To conserve computational resources, we first performed standalone feature extraction for the imaging modality of patients requiring subsequent training, and saved the extracted features as matrix files. As a result, the imaging modality model did not participate in parameter updates during subsequent training. Since the imaging features were derived using a pre-trained model and this model was not integrated into the full pipeline, no further parameter tuning was conducted for it.

To accommodate this design, the MDD-CoD1 model underwent full-parameter pre-training of the FT-Transformer, during which all subsequent layers—including the unimodal classification layer, multimodal fusion layer, and multimodal classification layer—were temporarily frozen. After the FT-Transformer pre-training phase, all of its layers were permanently frozen, and only the parameters of the unimodal classification layer, multimodal fusion layer, and final classification layer were updated during the training phase. The key hyperparameters are listed in the table below.

We employed a random split strategy for dataset partitioning, allocating 70% of the data to the training set and 30% to the test set. Detailed training hyperparameters are provided in the table below.

Table S6. MDD-CoD1 Hyperparameter

**Hyperparameter**	**RA**	**MN**	**CRC**	**CKD**
Input dim	768	768	1536	192
Epochs	400	300	300	200
Pretrain epochs	100	70	70	30
Optimizer	AdamW	AdamW	AdamW	AdamW
Learning rate	1e-4	1e-3	1e-3	1e-4
Decay weight	2e-3	1e-4	5e-4	1e-4

MDD-CoD2： For the MDD-CoD2 model, we adopted 4-fold cross-validation (K=4), dividing the original dataset into four subsets. In each iteration, three subsets were used for training and one for validation, with each subset serving as the validation set exactly once. The main evaluation metrics included AUROC, F1-score,

and Precision. Key hyperparameters are also summarized in the table.

Table S7. MDD-CoD2 Hyperparameter

**Hyperparameter**	**RA**	**MN**	**CKD**
Batch size	256	256	256
Epochs	1000	300	1000
Optimizer	AdamW	AdamW	AdamW
Learning rate	1e-3	1e-3	1e-4
Decay weight	2e-3	2e-3	1e-4
Scheduler	Steplr（70\0.85）	Steplr（50\0.85）	Steplr（70\0.85）
Memory using	7GB	7GB	7GB
Training time	6.68h	1.33h	6h
OverSampler	False	True	False

MDD-CoD3: Prior to training the MDD-CoD2 model, patient and medication features were comprehensively extracted using the preceding two-stage models and saved as matrix files. During model development, the dataset was randomly partitioned into 70% for training and 30% for testing. The specific training hyperparameters are listed in the table below.

Table S8. MDD-CoD3 Hyperparameter

**Hyperparameter**	**value**
Batch size	128
Input dim	256
hidden dim	512
output dim	128
Epochs	1000
Optimizer	Adam
Learning rate	1e-4
Decay weight	2e-4

Given the current version of the dataset, we found it unnecessary to use an additional validation set for model selection. This is due to the limited dataset size—mainly resulting from the difficulty of collecting sufficient data for rare or complex diseases. Allocating a separate validation set would reduce the size and diversity of the training set, potentially degrading model performance. Our dataset construction approach helps bridge the current data gap in this field and lays a solid foundation for future deployment and scalability.

Appendix 4 ：Evaluation of Indicators

The evaluation metrics employed in this study are standard indicators commonly used in deep learning-based prediction tasks. The corresponding formulas are provided below.

In classification model evaluation, True Positive (TP), True Negative (TN), False Positive (FP), and False Negative (FN) are fundamental performance indicators used to describe prediction accuracy and error types. Specifically, TP and TN represent the number of correctly predicted positive and negative samples, respectively, while FP and FN denote the number of incorrectly predicted positive and negative samples, respectively.

For the MDD-CoD1 model, the evaluation metrics included the Area Under the Receiver Operating Characteristic Curve (AUROC) and the Area Under the Precision-Recall Curve (AUPRC). AUROC is a broadly applicable metric across most prediction tasks, while AUPRC is particularly sensitive to class imbalance. Their combined use helps mitigate potential biases introduced by using a single metric under imbalanced conditions.

For the MDD-CoD2 model, we employed AUROC, F1-Score, and Precision. The F1-Score compensates for the limitations of AUROC and Precision when evaluating models on imbalanced datasets, with a particular focus on the model's ability to correctly classify minority classes.

For the MDD-CoD3 model, we used Hit@K as the evaluation metric. Hit@K is widely used in recommendation systems and information retrieval, and measures whether the ground-truth item appears within the top-K predicted items. The corresponding formulas are as follows:

$$\mathrm{TPR}=\frac{\mathrm{TP}}{\mathrm{TP+FN}} \quad (1)$$

$$\mathrm{FPR}=\frac{\mathrm{TP}}{\mathrm{TP+FN}} \quad (2)$$

$$\mathrm{AUROC}=\frac{1}{2}\sum_{i=1}^{n}(\mathrm{FPR}_i-\mathrm{FPR}_{i-1})(\mathrm{TPR}_i-\mathrm{TPR}_{i-1}) \quad (3)$$

$$P=\frac{TP}{TP+FP} \tag{4}$$

$$R=\frac{TP}{TP+FN} \tag{5}$$

$$AURRC=\frac{1}{2}\sum_{i=1}^{n}(R_i-R_{i-1})(P_i-P_{i-1}) \tag{6}$$

$$F1=\frac{2PR}{P+R}=\frac{2TP}{2TP+FP+FN} \tag{7}$$

For a single test sample, Hit@k is a binary metric: （8）

$$Hit@k=\begin{cases}1,\text{if the correct answer is among the top- k predictions}\\ 0,\text{otherwise}\end{cases}$$

The average Hit@k over a test set of N samples is computed as: （9）

$$Hit@k=\frac{1}{N}\sum_{i=1}^{N}Hit@k$$

Appendix 5：Comparative Advantages of Core Technologies in MDD-CoD

FT-Transformer's Superiority in MDD-CoD1. In the MDD-CoD1 model, for the medical diagnostic form data, we introduce FT-Transformer as an embedding encoder for laboratory indicators, aiming to leverage its global dependency modelling capability across feature columns. Taking the RA dataset as an example, the category-based features are pain, limitation of movement, stiffness, and swelling of joints; and the numerical features are CRP, ESR, RF, AST, and ALT. A priori, there is a strong relationship between symptoms such as joint pain, limitation of movement, stiffness, and swelling, and inflammatory indicators such as CRP and ESR, in which the stronger the inflammatory response, the more severe the patient's symptoms. Liver indicators such as rheumatoid factor (RF) and AST/ALT can reflect the activity or complications of rheumatic diseases. The joint modeling of these features through the embedding layer can fully reflect the clinical characteristics of patients.

Multi-attribute medication feature's Superiority in MDD-CoD2. Macro-pharmacological characteristics (Properties) of medications include the typical representative description of the text of the medications, specifically the Chinese medicine natures, tastes, and meridian tropisms, as well as the side effects of Western medicines to reflect their macro-pharmacological effects in the field of traditional Chinese medicine. Meridian tropisms refer to the effect of the medication on the five zang-organs and the six fu-organs, which builds up the scope of application and efficacy of the medication. Natures and tastes reflect the nature of the medication and the characteristics of its action. The efficacy corresponds to the therapeutic symptoms. This will serve as the more intuitive functional information of the medications. As the macroscopic pharmacological characteristics of western medications, the side effects of western medications stem from the interaction of their chemical components with the human body. The selection of this as a pharmacological feature of western medications in this study aims to portray the concept of the combination of Chinese and western medications to reduce toxicity. The medications' microscopic protein

network features (PPI) include the protein network composed of the drug target and its related proteins, aiming to construct a comprehensive protein network structural feature representation, focusing on the interaction network topology of the medications' target proteins in the biological system to analyze the target protein's pivot, modularity, and functional conduction pathway in the global protein interaction network. Medication micro-protein sequence characterization (Protein-Seq) contains the medication's target protein sequences that decode features of protein structure and function.

DeepWalk' s Superiority in Structure-Only PPI Network Embedding. In graph neural networks (GNNs), the absence of node features reduces neighborhood aggregation to mere degree distribution statistics, preventing the extraction of high-order semantic information and thereby diminishing the advantages typically offered by GNNs.

Metapath2Vec, which is specifically designed for heterogeneous graphs, relies on predefined metapaths (e.g., "protein-function-protein") to guide random walks. However, in this task, the existence of only a single edge type causes these metapaths to degenerate into simple repeated edge traversals, which ultimately leads to suboptimal performance.

LINE preserves local structural information by using a degree-based negative sampling strategy to optimize first-order (direct neighbor) and second-order (shared neighbor) node similarities. However, it tends to treat high-degree nodes as hubs or structural centers while overlooking the importance of critical bridge proteins, and thus fails to capture the global topological characteristics of protein-protein interaction (PPI) networks.

This study conducts comparative experiments among DeepWalk, Graph Neural Networks, Metapath2Vec, and LINE for network embedding. The experimental results demonstrate that DeepWalk achieves the best performance in embedding PPI features. The strength of DeepWalk lies in its strong compatibility with the homogeneous structure of PPI networks. The node co-occurrence distributions derived from random walks effectively encode the network's topology: short walks

are sufficient to cover most nodes while capturing both hub nodes and crucial bridge nodes, thus yielding superior topological representations.

Appendix 6: Model Generalizability Between Diseases and Hospitals

To demonstrate the model's (MDD-CoD1) generalizability, we pre-trained it using data from one disease and then fine-tuned it using data from another disease (25% of the total dataset) to validate its cross-disease transferability. As shown in Table S9, in most transfer scenarios, MDD-CoD1 outperformed baseline models trained on the full target-domain data. Notably, in the transfer from colorectal cancer (CRC) to chronic kidney disease (CKD), both of which share similar image modalities, MDD-CoD1 achieved up to a 15% improvement in AUROC. Even in a less favorable setting, such as CRC to rheumatoid arthritis (RA), the performance gap was kept within 3%. These results demonstrate the strong generalization ability of MDD-CoD1, with limited target-domain data, the fine-tuned pretrained model can surpass baseline models trained from scratch on full data. Most pre-trained models achieve performance surpassing the benchmark model trained on the full dataset of the target disease after fine-tuning with a small portion (25%) of the target disease data. Additionally, we provide the loss curve of MDD-CoD1 on the training set to explain the underlying mechanism of this improvement. As shown in Figure S5, across four datasets, the loss curves (e.g., the comparison between red and blue lines) exhibit lower training loss and faster convergence rates, indicating that the knowledge learned by MDD-CoD1 on source domain data facilitates learning for downstream tasks, thereby validating the model's generalizability.

Regarding the generalizability across hospitals and different diseases, we agree that further hierarchical analysis is needed, along with external validation across different hospitals and datasets. In fact, our data already covers multiple hospitals. Additionally, we plan to further expand and maintain this dataset in future research.

Table S9. Model performance comparison between transfer learning and full fine-tuning approaches.

（Source->Target）	AUROC↑	AUPRC↑

CRC->RA	0.9244（0.8989）	0.8137（0.7481）
CRC->CKD	0.6889（**0.7920**）	0.5630（**0.5708**）
MN->CRC	0.7231（**0.7736**）	0.8977（**0.9164**）
CKD->MN	0.7136（**0.7573**）	0.5940（**0.6650**）

Where “Source” refers to models trained on the complete dataset of the source disease, while “Target” refers to models trained based on the source disease. X(Y) denotes the test set accuracy on the original dataset (test set accuracy after small-sample transfer learning).

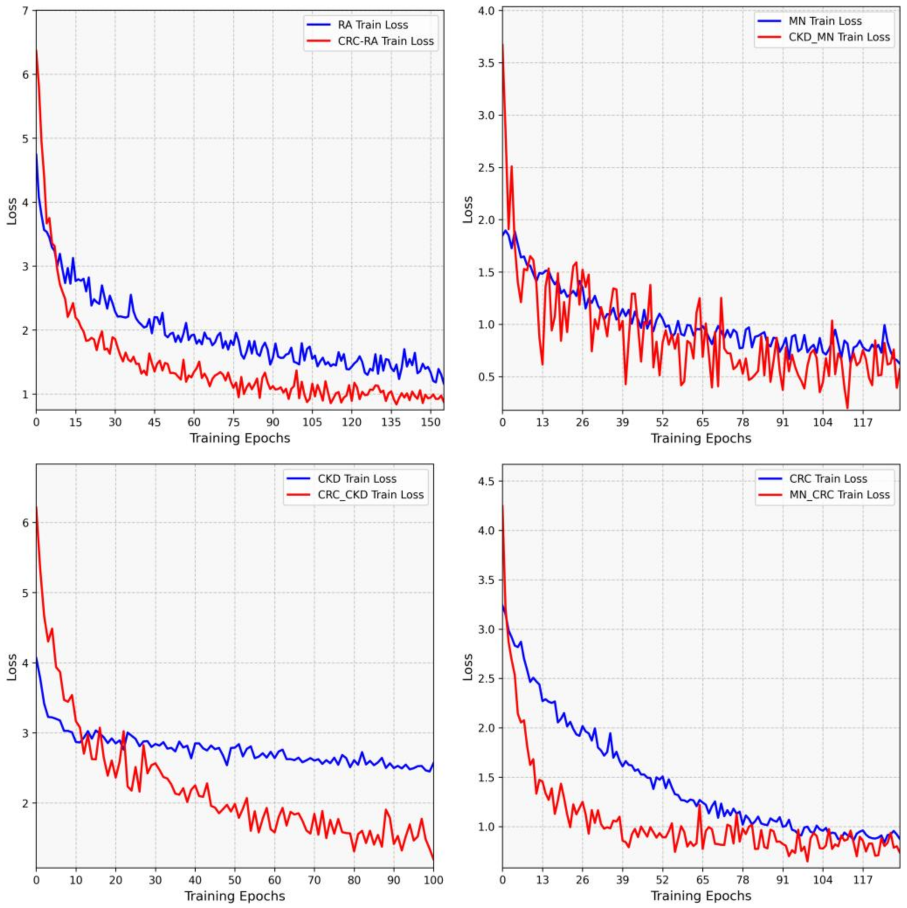

Figure S5: MDD-CoD demonstrates excellent generalization performance on multiple datasets. The blue curve represents the original training loss curve, while the red curve represents the training loss curve after small-sample transfer learning. On all four datasets, MDD-CoD demonstrates lower training loss and faster convergence speed.

Appendix 7: MDD-CoD1 Interpretability Study

We used cosine similarity, a standard metric for cross-modal alignment research, to construct a modal feature interaction heatmap for interpretability research. By analyzing 13 random training samples from two datasets (CRC and MN), as shown in Figure S6: During the initial training phase, cross-modal similarity was generally low (CRC: -0.0363, MN: -0.0772). After training, the multi-modal features of both datasets showed an increase in average similarity (CRC: +0.1285, MN: +0.1602). This change confirms that the model has successfully established foundational semantic association capabilities, effectively validating the efficacy of MDD-CoD in cross-modal alignment tasks.

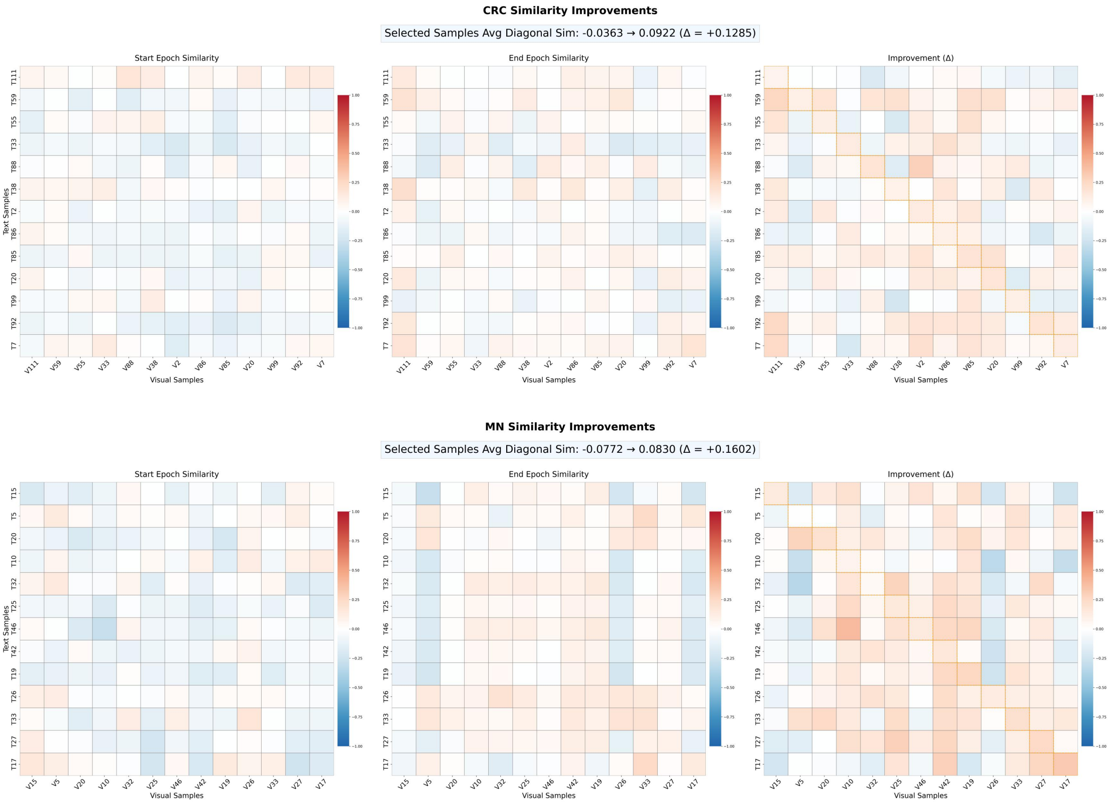

Figure S6: Cross-modal feature alignment heatmap on the CRC and MN datasets. A modal interaction heatmap based on cosine similarity, showing the evolution of cross-modal alignment from initial training (left) to final convergence (right) for 13 random samples. Blue tones (-0.1 to 0) indicate weak modal correlation, red tones (0

to 0.2) indicate strong cross-modal alignment.

Appendix 8: Abbreviation of Medication

Medication	Abbreviation of Medication
Compound Nephritis Tablets	CNT
Zingiberis Rhizoma Recens	Rhiz. Zing
Centellae Herba	C. Herba
Jujubae Fructus	J. Fructus
Lycii Fructus	L. Fructus
Rubiae Radix Et Rhizoma	R. Radix
Aspirin	Rad. Rub.
Cordyceps cicadae	C. cicadae
Aucklandiae Radix	A. Radix
Terazosin	TER
Platycodonis Radix	P. Radix
Poriae Cutis	P. Cutis
Rhapontici Radix	R. Radix
Irbesartan	IRB
Dianthi Herba	D. Herba
Pitavastatin	PTV
Lophatheri Herba	L. Herba
Amlodipine Besilate	AML-B
Rosuvastatin	Rosuv.
Cyperi Rhizoma	Rhiz. Cyperi
Dioscoreae Hypoglaucae Rhizoma	Rhiz. Diosc. Hypogl.
Microcos Paniculata	Microcos
Poria	Por.
Pheretima	Pher.
Hemocoagulase Injection	Inj. Hemocoag
Forsythiae Fructus	Fruct. Forsyth.
Valsartan And Amlodipine Tablets A	Tab. Vals. & Amlod.
Bambusae Caulis In Taenias	Caul. Bamb.
Pogostemonis Herba	Herb. Pogost.
Salviae Miltiorrhizae Radix Et Rhizoma	Rad. et Rhiz. Salviae
Sanqi Oral Liquid	Liq. Oral. Notogins
Jinshuibaopian	JSB
Paeoniaeradix Rubra	Rad. Paeon. Rub.
Polygoni Multiflori Caulis	Caul. Polyg. Mult.
Aluminum Magnesium Carbonate And Aspirin	Alum. Mag. Carb. & Asp.
Anemarrhenae Rhizoma	Rhiz. Anemarrh.

Scutellariae Radix	Rad. Scutell.
Terazosin Hydrochloride	Teraz. HCl
Fritillariae Thunbergii Bulbus	Bulb. Fritill. Thunb
Euryales Semen	Sem. Euryal.
Imperatae Rhizoma	Rhiz. Imper.
Clematidis Radix Et Rhizoma	Rad. et Rhiz. Clemat.
Pitavastatin	Pitav.
Chuanxiong Rhizoma	Rhiz. Chuanx
Alismatis Rhizoma	Rhiz. Alism.
Polyporus	Polypor.
Polyene Phosphatidylcholine	Phosph. Polyen.
Semen Armeniacae Amarum	Sem. Armen. Am.
Glycyrrhizae Radix Et Rhizoma Praeparata Cum Melle	Rad. et Rhiz. Glycyrrh. Praep.
Zingiberis Rhizoma	Rhiz. Zingib.
Scleromitrion Diffusum	Scleromit.
Methylprednisolone	Methylpred.
Nelumbinis Stamen	Stam. Nelumb.
Carvedilol	Carved.
Torsemide	Torsem.
Corni Fructus	Fruct. Corni
Cyclophosphamide	Cyclophosph.
Dendrobium With Fruits	Dendrob. c. Fruct.
Bupleuri Radix	Rad. Bupl.
Fluvastatin	Fluvast.
Corni Fructus	Fruct. Corni
Cuscutae Semen	Sem. Cusc.
Huangkui Jiaonang	HKJN
Atractylodis Macrocephalae Rhizoma	Rhiz. Atract. Macro.
Recombinant Human Erythropoietin Polysaccharide-Iron Complex Capsules	Caps. EPO-PIC
Complexus Polysaccharidi-Ferrosus	CPF
Low Molecular Weight Heparin	Heparin. LMWH
Furosemide Tablets	Tab. Furos.
Compound Ketoacid Tablets	Tab. Ketoacid. Co.
Eucommiae Cortex Praeparatus	Cort. Eucomm. Praep.
Low Molecular Weight Heparin Calcium	Heparin. Calc. LMWH
Bisoprolol Fumarate	Bisoprol. Fum.
Calcitriol	Calcitriol.
Gliclazide	Gliclaz.
Acarbose	Acarb.
Albiziae Flos	Fl. Albiz.
Codonopsis Radix	Rad. Codon.

Spironolactone	Spironol.
Calcium Carbonate D3	Calc. Carb. D3
Asari Radix Et Rhizoma	Rad. et Rhiz. Asari
Talcum	Talc.
Calcium Carbonate D3	Calc. Carb. D3
Gentianae Macrophyllae Radix	Rad. Gent. Macroph
Chaenomelis Fructus	Fruct. Chaenom.
Cinnamomi Ramulus	Ram. Cinnam.
Fluvastatin	Fluvast.
Atractylodis Rhizoma	Rhiz. Atract
Diospyros Iotus Linn	Diosp. Lot.
Glycyrrhizae Radix Et Rhizoma	Rad. et Rhiz. Glycyrrh.
Polygoni Cuspidati Rhizoma Et Radix	Rhiz. et Rad. Polyg. Cusp.
Atractylodis Macrocephalae Rhizoma	Rhiz. Atract. Macro.
Raphani Semen	Sem. Raph.
Oryzae Fructus Germinatus	Fruct. Oryz. Germ.
Asini Corii Colla	Coll. Cor. Asin.
Ostreae Concha	Conch. Ostr.
Schisandrae Chinensis Fructus	Fruct. Schisand. Chin.
Pyrrosiae Folium	Fol. Pyrros.
Uremic Clearance Granule	Gran. Urem. Clear.
Tacrolimus	Tacrol.
Lonicerae Japonicae Flos	Fl. Lonic. Jap.
Coicis Semen Tostum	Sem. Coic. Tost.
Corydalis Decumbentis Rhizoma	Rhiz. Coryd. Decumb.
Compound A-Keto Acid	A-Keto Acid Co.
Magnoliae Officinalis Cortex	Cort. Magn. Off.
Amomi Fructus Rotundus	Fruct. Amom. Rot.
Linagliptin	Linaglipt.
Entecavir	Entecav.
Moutan Cortex	Cort. Moutan
Uncariae Ramulus Cum Uncis	Ram. Unc. c. Unc.
Lycopi Herba	Herb. Lycop
Febuxostat	Febux.
Eucommiae Cortex	Cort. Eucomm.
Persicae Semen	Sem. Pers.
Dendrobii Caulis	Caul. Dendrob.
Gardeniae Fructus	Fruct. Gard.
Saposhnikoviae Radix	Rad. Saposhn.
Cassiae Semen	Sem. Cass.
Curcumae Rhizoma	Rhiz. Curc.
Dioscoreae Nipponicae Rhizoma	Rhiz. Diosc. Nipp.
Fructus Hordei Germinatus	Fruct. Hord. Germ.

Aconitum Carmichaelii Preparatum	Acon. Carm. Prep.
Carvedilol Tablets	CT
Citri Reticulatae Pericarpium	Pericarp. Cit. Ret.
Hedysari Radix	Rad. Hedys.
Hordei Fructus Germinatus	Fruct. Hord. Germ.
Angelicae Sinensis Radix	Rad. Ang. Sin.
Levothyroxine	Levothyrox.
Polygoni Multiflori Radix Praeparata	Rad. Polyg. Mult. Praep.
Sinapis Semen	Sem. Sinap.
Rosae Laevigatae Fructus	Fruct. Ros. Laevig.
Valsartan	Valsart.
Atorvastatin	Atorvast.
Polygoni Avicularis Herba	Herb. Polyg. Avic.
Eclipta Prostrata	Eclipt.
Epimedii Folium	Fol. Epim.
Spatholobi Caulis	Caul. Spath.
Perillae Fructus	Fruct. Perill.
Polygonati Rhizoma	Rhiz. Polygon.
Metformin Hydrochloride Tablets	MHC
Puerariae Lobatae Radix	Rad. Puer. Lob.
Dalteparin	DNS
Paeoniae Radix Alba	Rad. Paeon. Alb.
Phellodendri Chinensis Cortex	Cort. Phell. Chin.
Shenyan Kangfu Pian	SKP
Losartan Potassium	LP
Nelumbinis Semen	Sem. Nelumb.
Amomi Fructus	Fruct. Amom.
Astragali Radix Praeparata Cum Melle	Rad. Astrag. Praep. c. Mel.
Frusemide	Frusem.
Rehmannia Glutinosa	Rehmann.
Erythropoietin	EPO
Rabeprazole	Rabepraz.
Pinelliae Rhizoma Praeparatum	Rhiz. Pinell. Praep.
Panax Notoginseng Powder	Pulv. Panax Notog.
Aurantii Fructus	Fruct. Aurant.
Zea Mays Stigmas	Stigm. Zeae
Corydalis Rhizoma	Rhiz. Coryd.
Arisaema Cum Bile	Aris. c. Bil.
Cistanches Herba	Herb. Cistan.
Pseudostellariae Radix	Rad. Pseudostell.
Human Albumin	Alb. Hum.
Coicis Semen	Sem. Coic.
Arctii Fructus	Fruct. Arct.

Astragali Radix	Rad. Astrag.
Chrysanthemi Flos	Fl. Chrysanth.
Aspirin Enteric-Coated Tablets	AEC
Liquidambarisfructus	Fruct. Liquidamb.
Haliotidis Concha	Conch. Haliot.
Leonuri Herba	Herb. Leon.
Rheum Palmatum L.	Rheum palm.
Gastrodiae Rhizoma	Rhiz. Gastrod.
Valsartan And Amlodipine Tablets	Tab. Vals. & Amlod.
Colchicine	Colchic.
Schizonepetae Spica	Spic. Schizon.
Rehmanniae Radix Praeparata	Rad. Rehmann. Praep.
Smilacis Glabrae Rhizoma	Rhiz. Smilac. Glabr.
Fluvastatin Sodium	Fluvast. Na
Rhei Radix Et Rhizoma	Rad. et Rhiz. Rhei
Sepiae Endoconcha	Endoconch. Sep.
Human Immunoglobulin	Ig Hum.
Asteris Radix Et Rhizoma	Rad. et Rhiz. Aster
Plantaginis Herba	Herb. Plantag.
Polysaccharide Iron	Fe Polysac.
Ligustri Lucidi Fructus	Fruct. Ligust. Luc.
Arecae Pericarpium	Pericarp. Arec.
Trichosanthis Radix	Rad. Trichos.
Plantaginis Semen	Sem. Plantag.
Coptidis Rhizoma	Rhiz. Copt.
Achyranthis Bidentatae Radix	Rad. Achyr. Bident.
Ficus Simplicissima	Ficus simpl.
Cornus Officinalis With Salt	Corn. off. c. Sal.
Ophiopogonis Radix	Rad. Ophiopog.
Notoginseng Radix Et Rhizoma	Rad. et Rhiz. Notogins.
Armeniacae Semen Amarum	Sem. Armen. Am.
Atorvastatincalcium	Atorvast. Ca
Metformin Hydrochloride Tablets	MHC
Epo	EPO
Sodium Zirconium Cyclosilicate	Zr Cyclosil. Na
Corni Fructus	Fruct. Corni
Cirsii Herba	Herb. Cirs.
Metoprolol	Metoprol.
Rubi Fructus	Fruct. Rubi
Yishen Huashi Granules	YSHG
Menthae Haplocalycis Herba	Herb. Menth. Haploc.
Folic Acid Tablets	FAT
Torasemide Tablets	TOR

Morindae Officinalis Radix	Rad. Morind. Off.
Monascus Purpureus	Monasc. purp.
Dioscoreae Rhizoma	Rhiz. Diosc.
Sodium Bicarbonate	SBC
Iguratimod Tablets	IGU Tab.
Lilii Bulbus	Lil. Bulb.
Massae Medicatae Fermentatae Tostum	M. Medic. Ferr. Tost.
Gardeniae Fructus Praeparatus	Gard. Fruct. Praep.
Phellodendri Amurensis Cortex	Phell. Amur. Cort.
Ephedrae Herba	Eph. Herb.
Ginseng Radix et Rhizoma	Gins. Rad. Rhiz.
Baricitinib Tablets	Baricitinib Tab.
Methotrexate Tablets	MTX Tab.
Methylprednisolone Tablets	Methylprednisolone Tab.
Paridis Rhizoma	Parid. Rhiz.
Prednisone Acetate Tablets	PA
Cicadae Periostracum	Cicad. Perios.
KunXianJiaoNang	KXJN
Leflunomide Tablets	LEF
Tinosporae Caulis	Tinos. Caul.
Angelicae Pubescentis Radix	Ang. Pub. Rad.
Taxilli Herba	Tax. Herb.
Citri Reticulatae Pericarpium	Cit. Ret. Pericarp.
Millettiae Speciosae Radix	Mill. Spec. Rad.
Coriolus	Coriol.
Scorpio	Scorp.
Angelicae Dahuricae Radix	Ang. Dah. Rad.
Erigerontis Herba	Erig. Herb.
LeiGongTenDuoGanPian	LGTDGP
Curcumae Longae Rhizoma	Curc. Long. Rhiz.
Stephaniae Tetrandrae Radix	Steph. Tet. Rad.
Mori Ramulus	Mori Ramulus
Siegesbeckiae Herba	Sieg. Herb.
ZhengQingFengTongNingHuanShiPian	ZQFTNHSP
Cibotii Rhizoma	Cib. Rhiz.
Drynariae Rhizoma	Dryn. Rhiz.
Dipsaci Radix	Dip. Rad.
BaiShaoZongGanJiaoNang	BSZGJN
Cannabis Semen	Cann. Sem.
Kochiae Fructus	Koch. Fruct.
Glehniae Radix	Glehn. Rad.
Sophorae Flavescentis Radix	Soph. Flav. Rad.
Perillae Folium	Perill. Fol.

Dictamni Cortex	Dict. Cort.
Cynanchi Paniculati Radix et Rhizoma	Cyn. Pan. Rad. Rhiz.
BiQiJiaoNang	BQJN
Trachelospermi Caulis	Trache. Caul.
Poria Cum Radix Pini	Por. Cum Rad. Pin.
Notopterygii Rhizoma et Radix	Notop. Rhiz. Rad.
Glechomae Herba	Glech. Herb.
Recombinant Human Tumor Necrosis Factor-α Receptor II: IgG Fc Fusion Protein	TNFR-Fc
Aurantii Fructus Immaturus	Aur. Fruct. Imm.
Polygalae Radix Praeparata	Polyg. Rad. Praep.
Dendrobii Nobile Caulis	Dendrob. Nob. Caul.
Adalimumab	ADA
Citri Sarcodactylis Fructus	Cit. Sarc. Fruct.
Os Draconis	Os Drag.
Eriobotryae Folium	Eriob. Fol.
Cayratiae Herba	Cayr. Herb.
Benincasae Exocarpium	Ben. Exocarp.
Etoricoxib Tablets	ETX
Zaocys	Zaoc.
Cyclosporine Soft Capsules	CsA
Erythrinae Cortex	Eryth. Cort.
Celecoxib Soft Capsules	Celecoxib Cap.
Achyranthis Bidentatae Radix Praeparata Cum Vino	Achyr. Bid. Rad. Praep. Vin.
Piperis Kadsurae Caulis	Pip. Kad. Caul.
Arcae Concha Praeparata	Arc. Conch. Praep.
Luffae Fructus Retinervus	Luff. Fruct. Ret.
Cimicifugae Rhizoma	Cimic. Rhiz.
Artemisiae Scopariae Herba	Artem. Scop. Herb.
Eupatorii Herba	Eupat. Herb.
Crataegus Pinnatifida	Crataeg. Fruct. Tost.
Galli Gigerii Endothelium Corneum	Gall. Gig. Endoth. Corn.
Diclofenac Sodium Sustained - release Tablets	DSST
Tofacitinib Tablets	TFC
Carthami Flos	Carth. Flos
Ziziphi Spinosae Semen	Ziziph. Spin. Sem.
Albiziae Cortex	Alb. Cort.
Nidus Vespae	Nid. Vesp.
Lonicerae Caulis	Lon. Caul.
Hydroxychloroquine Sulfate Tablets	HCQ
Lycopodii Herba	Lycop. Herb.
WangBiJiaoNang	WBJN

Glechomae Longitubae Herba	Glech. Long. Herb.
Sinomenii Caulis	Sinom. Caul.
Phyllanthi Fructus	Phyll. Fruct.
Aurantii Fructus Immaturus Praeparatus Cum Braisiae	Aur. Fruct. Imm. Praep. Brais.
Gleditsiae Spina	Gled. Spin.
Crataegi Fructus	Crataeg. Fruct.
Massae Medicatae Fermentatae	M. Medic. Ferr.
Myristicae Semen Praeparatum Cum Braisiae	Myrist. Sem. Praep. Brais.
Psoraleae Fructus	Psoral. Fruct.
Momordicae Fructus	Momord. Fruct.
Gossampini Flos	Goss. Flos
Phragmitis Rhizoma	Phrag. Rhiz.
Mume Fructus	Mum. Fruct.
Compound Glycyrrhizin Tablets	CGT
Artemisiae Argyi Folium	Artem. Arg. Fol.
Micae Lapis Aureus	Mic. Lap. Aur.
Diammonium Glycyrrhizinate Enteric - coated Capsules	DGEC
Aconiti Lateralis Radix Praeparata	Acon. Lat. Rad. Praep.
Periplocae Cortex	Peripl. Cort.
Bombyx Batryticatus	Bomb. Batryt.
Atractylodis Macrocephalae Rhizoma Praeparatum Cum Braisiae	Atr. Macro. Rhiz. Praep. Brais.
Citri Grandis Exocarpium Rubrum	Cit. Grand. Exocarp. Rub.
Sarcandrae Herba	Sarc. Herb.
Iris domestica	I. domestica
Trichosanthes kirilowii (Cortex)	T. kirilowii
Commiphora myrrha (Vinegar-processed)	C. myrrha
Zanthoxylum bungeanum	Z. bungeanum
Boswellia carterii	B. carterii
Clematis armandii (Caulis)	C. armandii
Curculigo orchioides	C. orchioides
Prinsepia uniflora	P. uniflora
Eupolyphaga sinensis	E. sinensis
Pittosporum glabratum	P. glabratum
Gypsum Fibrosum	G. Fibrosum
Mycophenolate Mofetil Capsules	MMF
Coix lacryma-jobi var. ma-yuen (Bran-fried)	C. lacryma-jobi var. ma-yuen
Triticum aestivum var. aestivum (Blighted)	T. aestivum var. aestivum
Tribulus terrestris	T. terrestris
Oroxylum indicum	O. indicum
Psoralea corylifolia (Salt-processed)	P. corylifolia

Lycium chinense	L. chinense
Stemona sessilifolia	S. sessilifolia
Ephedra sinica (Honey-processed)	E. sinica
Aster tataricus (Honey-processed)	A. tataricus
Tussilago farfara	T. farfara
Schizonepeta tenuifolia	S. tenuifolia
Peucedanum praeruptorum	P. praeruptorum
Oryza sativa (Glutinous rice root)	O. sativa
Terminalia chebula	T. chebula
Cynanchum stauntonii	C. stauntonii
Diospyros kaki (Persistent calyx)	D. kaki
Arisaema erubescens (Processed)	A. erubescens
Prunus persica (Parched seed)	P. persica
Zanthoxylum nitidum	Z. nitidum
Rosa laevigata (Fructus, fleshy part)	R. laevigata
Endothelium Corneum Gigeriae Galli (Stir-fried)	E. Corneum
Scrophularia ningpoensis	S. ningpoensis
Cinnamomum cassia	C. cassia
Morus alba (Cortex)	M. alba
Vitex negundo var. cannabifolia (Fructus)	V. negundo
Ganoderma capense	G. capense
Polygonatum odoratum	P. odoratum
Acorus tatarinowii	A. tatarinowii
Semen Sojae Praeparatum (Fermented soybean)	S. Sojae
Curcuma phaeocaulis	C. phaeocaulis
Sparganium stoloniferum	S. stoloniferum
Dolichos lablab (Flos)	D. lablab
Magnolia officinalis (Flos)	M. officinalis
Myristica fragrans	M. fragrans
Polygonum perfoliatum	P. perfoliatum
Morus alba (Ramulus)	M. alba
Agkistrodon	A. spp.
Polygala tenuifolia (Honey-processed)	P. tenuifolia
Prunella vulgaris	P. vulgaris
Aconitum carmichaelii (Processed)	A. carmichaelii
Magnolia biondii	M. biondii
Turpinia arguta (Folium)	T. arguta
Xanthium sibiricum (Fructus)	X. sibiricum
Triamcinolone Acetonide Acetate Injection	TAA
Sterculia lychnophora	S. lychnophora
Saussurea involucrata	S. involucrata
Corydalis yanhusuo (Vinegar-processed)	C. yanhusuo
Panax ginseng (Folium)	P. ginseng

Eriobotrya japonica (Honey-processed Folium)	E. japonica
Oryza sativa (Germinatus)	O. sativa
Polygonum chinense	P. chinense
Platycladus orientalis (Semen)	P. orientalis
Linum usitatissimum (Semen)	L. usitatissimum
Concha Ostreae (Praeparata)	C. Ostreae
Taraxacum mongolicum	T. mongolicum
Caesalpinia sappan (Lignum)	C. sappan
Perilla frutescens (Caulis)	P. frutescens
Dimocarpus longan (Arillus)	D. longan
Chimonanthus praecox (Flos)	C. praecox
Atractylodes macrocephala (Stir-fried)	A. macrocephala
Dolichos lablab (Semen)	D. lablab
Os Draconis Praeparata	O. Draconis
Atractylodes macrocephala (Stir-fried with clay)	A. macrocephala
Cervus nippon (Cornu, powdered)	C. nippon
Phellodendron amurense (Salt-processed)	P. amurense
Desmodium styracifolium	D. styracifolium
Tetrapanax papyrifer (Medulla)	T. papyrifer
Agrimonia pilosa	A. pilosa
Sanguisorba officinalis (Carbonised)	S. officinalis
Ziziphus jujuba var. spinosa (Stir-fried Semen)	Z. jujuba
Nelumbo nucifera (Folium)	N. nucifera
Morus alba (Folium)	M. alba
Dicliptera chinensis	D. chinensis
Vitex trifolia (Fructus)	V. trifolia
Cynomorium songaricum	C. songaricum
Curcuma aromatica	C. aromatica
Santalum album	S. album
Ligusticum sinense	L. sinense
Citrus aurantium (Bran-fried Exocarpium)	C. aurantium
Chinemys reevesii (Vinegar-processed)	C. reevesii
Golimumab	GLM
Stauntonia chinensis	S. chinensis
Scolopendra subspinipes	S. subspinipes
Bombyx mori Faeces	B. mori
Sulfasalazine Enteric-coated Tablets	SSZ
Asparagus cochinchinensis	A. cochinchinensis
Zhengqing Fengtongning Injection	ZQFTN
Inula japonica	I. japonica
Haematitum	H. spp.
Arca subcrenata	A. subcrenata
Trichosanthes kirilowii (Semen)	T. kirilowii

Moghania philippinensis	M. philippinensis
Phellodendron amurense (Stir-fried)	P. amurense
Chinemys reevesii (Carapax)	C. reevesii
Etanercept Injection	ETN
Alpinia oxyphylla	A. oxyphylla
Polysaccharide-Iron Complex Capsules	PIC
Sanguisorba officinalis	S. officinalis
Platycladus orientalis	P. orientalis
Lindera aggregata	L. aggregata
Raphanus sativus (Tostus)	R. sativus
Luobitong Pian	L. Bitong
Bambusae concretio silicea	B. concretio
Croton crassifolius	C. crassifolius
Semiliquidambar cathayensis	S. cathayensis
Ardisia gigantifolia	A. gigantifolia
Pinellia ternata (Praeparata)	P. ternata
Acorus tatarinowii	A. tatarinowii
Fagopyrum dibotrys	F. dibotrys
Pregabalin Capsules	PGB
Cremastra appendiculata	C. appendiculata
Ursodeoxycholic Acid Capsules	UDCA
Methylprednisolone	MP
Prednisone	PDN
Cyclosporine Capsules	CYC
Metoprolol	MET
Alfacalcidol	ALF
Scutellaria barbata	S. barbata
Lithospermum erythrorhizon	L. erythrorhizon
Valsartan Capsules	VAL
Juncus effusus	J. effusus
Hydrochlorothiazide	HCTZ
Mel	Mel
Sophora japonica	S. japonica
Isatis indigotica	I. indigotica
Panax quinquefolius	P. quinquefolius
Trionyx sinensis (Praeparata)	T. sinensis
Typha angustifolia	T. angustifolia
Pulsatilla chinensis	P. chinensis
Dioscorea hypoglauca	D. hypoglauca
Magnetitum	Magnetitum
Vigna umbellata	V. umbellata
Cirsium japonicum	C. japonicum
Pteris multifida	P. multifida

Pumex	Pumex
Euonymus alatus	E. alatus
Sargassum fusiforme	S. fusiforme
Citrus reticulata (Semen)	C. reticulata
Oryza sativa	O. sativa
Tripterygium hypoglaucum	T. hypoglaucum
Ganoderma lucidum	G. lucidum
Litchi chinensis (Semen)	L. chinensis
Nelumbo nucifera (Plumula)	N. nucifera
Pyrola calliantha	P. calliantha
Fossilia Dentis Mastodi	F. Dentis
Rosa rugosa	R. rugosa
Lasiosphaera fenzlii	L. fenzlii
Nelumbo nucifera (Nodus)	N. nucifera
Bubali Cornu	B. Cornu
Punica granatum (Pericarpium)	P. granatum
Massa Medicata Fermentata	M. Fermentata
Brainea insignis	B. insignis
Cnidium monnieri	C. monnieri
Viola philippica	V. philippica
Areca catechu	A. catechu
Bletilla striata	B. striata
Rehmannia glutinosa	R. glutinosa
Ilex asprella	I. asprella
Trogopterus xanthipes (Faeces)	T. xanthipes
Allium macrostemon	A. macrostemon
Alpinia oxyphylla	A. oxyphylla
Mycophenolate Mofetil	MMF
Rabeprazole Sodium Enteric-coated Capsules	RAB
Allisartan Isoproxil	ALS
Insulin Lispro Protamine Mixed Suspension	ILP
Belimumab	BEL
Metformin	MET
Azathioprine	AZA
Niaodukang Heji	NDK
Repaglinide	RPG
Felodipine	FEL
Aconitum carmichaelii (Praeparata)	A. carmichaelii
Clopidogrel	CLO
Bailing Jiaonang	B. Jiaonang
Calcium Dobesilate	CaD
Nifedipine	NIF
Vitamin B Complex	VBC

Amlodipine	AML
Alendronate Sodium	ALE
Liniu Heji	L. Heji
Ezetimibe	EZE
Dapagliflozin	DAPA
Olmesartan Medoxomil	OLM
Fenofibrate	FEN
Dulaglutide	DULA
Lanthanum Carbonate	LAN
Tolvaptan	TOLV
Finerenone	FIN
Sacubitril/Valsartan Sodium	SAC/VAL
Canagliflozin	CANA
Insulin Lispro Protamine	ILP
Cinacalcet	CIN
Losartan Potassium/Hydrochlorothiazide	LOS/HCTZ
Callicarpa nudiflora	C. nudiflora
Human Albumin (20%)	HA
Esomeprazole	EZO
Benazepril	BZP
Irbesartan Tablets	IRB
Irbesartan and Hydrochlorothiazide Tablets	IRB/HCTZ
Empagliflozin Tablets	EMPA
Entecavir Tablets	ETV
Febuxostat Tablets	FEB
Fluvastatin	FLV
Calcium Tablets	Calcium Tablets
Heparin Sodium	HP
Pueraria lobata (Willd.) Ohwi	P. lobata
Metoprolol Tartrate	MTP
Canagliflozin Acetate Tablets	CANA
Canagliflozin Tablets	CANA
Entecavir Maleate Tablets	ETV
Calcium Carbonate and Vitamin D3 Tablets	CCDD3
Lepidium apetalum Willd.	TOR
Benazepril Hydrochloride Tablets	BZP
Prunus armeniaca L.	P. armeniaca
Benazepril Hydrochloride Tablets	BZP
YiLing KouFuYe	YLKFY
Cordyceps Militaris Mycelium Powder Capsule	CMPC
Zhenqi Fuzheng Tablet	ZQFZT
